# Epigenome environment interactions accelerate epigenomic aging and unlock metabolically restricted epigenetic reprogramming in adulthood

**DOI:** 10.1038/s41467-020-15847-z

**Published:** 2020-05-08

**Authors:** Lindsey S. Treviño, Jianrong Dong, Ahkilesh Kaushal, Tiffany A. Katz, Rahul Kumar Jangid, Matthew J. Robertson, Sandra L. Grimm, Chandra Shekar R. Ambati, Vasanta Putluri, Aaron R. Cox, Kang Ho Kim, Thaddeus D. May, Morgan R. Gallo, David D. Moore, Sean M. Hartig, Charles E. Foulds, Nagireddy Putluri, Cristian Coarfa, Cheryl Lyn Walker

**Affiliations:** 1 Center for Precision Environmental Health, Baylor College of Medicine, Houston, TX 77030 USA; 2 Department of Molecular and Cellular Biology, Baylor College of Medicine, Houston, TX 77030 USA; 3 Division of Health Equities, Department of Population Sciences, City of Hope, Duarte, CA 91010 USA; 4 Advanced Technology Cores, Baylor College of Medicine, Houston, TX 77030 USA; 5 Department of Medicine, Baylor College of Medicine, Houston, TX 77030 USA; 6 Department of Pediatrics, Baylor College of Medicine, Houston, TX 77030 USA; 7 Dan L. Duncan Comprehensive Cancer Center, Baylor College of Medicine, Houston, TX 77030 USA; 8 Department of Molecular and Human Genetics, Baylor College of Medicine, Houston, TX 77030 USA

**Keywords:** Genome-wide analysis of gene expression, Epigenetics

## Abstract

Our early-life environment has a profound influence on developing organs that impacts metabolic function and determines disease susceptibility across the life-course. Using a rat model for exposure to an endocrine disrupting chemical (EDC), we show that early-life chemical exposure causes metabolic dysfunction in adulthood and reprograms histone marks in the developing liver to accelerate acquisition of an adult epigenomic signature. This epigenomic reprogramming persists long after the initial exposure, but many reprogrammed genes remain transcriptionally silent with their impact on metabolism not revealed until a later life exposure to a Western-style diet. Diet-dependent metabolic disruption was largely driven by reprogramming of the Early Growth Response 1 (EGR1) transcriptome and production of metabolites in pathways linked to cholesterol, lipid and one-carbon metabolism. These findings demonstrate the importance of epigenome:environment interactions, which early in life accelerate epigenomic aging, and later in adulthood unlock metabolically restricted epigenetic reprogramming to drive metabolic dysfunction.

## Abstract (web summary)

Early life exposure to environmental stressors, including endocrine disrupting chemicals (EDCs), can impact health later in life. Here, the authors show that neonatal EDC exposure in rats causes epigenetic reprogramming in the liver, which is transcriptionally silent until animals are placed on a Western-style diet.

## Introduction

Environmental exposures during early life exert a profound influence on developing organs, which can affect health across the life-course, and even transgenerationally^1–4^. The adverse health impact of these exposures is thought to be mediated by reprogramming of normal physiologic responses, and forms the basis of the developmental origins of health and disease (DOHaD) paradigm^5,6^. Fetal over- or under-nutrition has been linked to metabolic dysfunction in adulthood and increased risk for metabolic diseases including obesity, diabetes and metabolic syndrome^1,7,8^. Besides nutritional stressors, early-life exposures to environmental chemicals, including endocrine-disrupting chemicals (EDCs), can influence health and disease susceptibility across the life-course.

EDCs are defined as exogenous chemicals, or mixture of chemicals, that interfere with hormone action^9^ and many have been shown to impact metabolic function, and increase disease risk in metabolic organs such as the liver^10,11^. Recently, the epigenetic machinery has emerged as a target for EDCs and other environmental exposures^12^. When this machinery is perturbed early in life, the resulting epigenetic alterations can persist long after the initial environmental insult (often referred to as developmental reprogramming)^13,14^. Accordingly, research on the causes of the epidemic rise in metabolic diseases has expanded beyond genetics, over-nutrition and energy expenditure to include the role of early-life EDC exposures^10,15–19^. However, little is known about what determines vulnerability to early-life exposures, or specific targets and pathways linking developmental reprogramming by early-life exposures to later-life metabolic dysfunction.

To understand how an early-life environmental exposure could drive adult metabolic dysfunction, we performed longitudinal epigenomic, transcriptomic and metabolomic profiling across the life-course after an early-life exposure to the prototypical EDC, bisphenol A (BPA). We report here that exposure to this EDC during a key developmental window, when the rodent liver is transitioning from a hematopoietic to a metabolic organ, induced epigenomic reprogramming by hijacking age-related epigenomic plasticity at specific genes and chromatin states in the neonatal liver to accelerate acquisition of an adult epigenetic signature. Although persistent into adulthood, much of this reprogramming remained transcriptionally silent until a later-life challenge with a Western-style diet high in fat, fructose, and cholesterol, which disrupted metabolic function and caused a significant elevation in serum cholesterol and lipids. Mechanistically, liver metabolic dysfunction was driven by EDC-induced epigenetic reprogramming of gene expression, signaling, and production of metabolites linked to cholesterol, lipid and one-carbon metabolism. These findings reveal that plasticity of the developing epigenome creates a vulnerability to reprogramming by environmental exposures, which may cause accelerated epigenomic aging and metabolic dysfunction conditional on later-life diet.

## Results

### Neonatal EDC exposure reprograms lipid metabolism

**Fig. 1:**
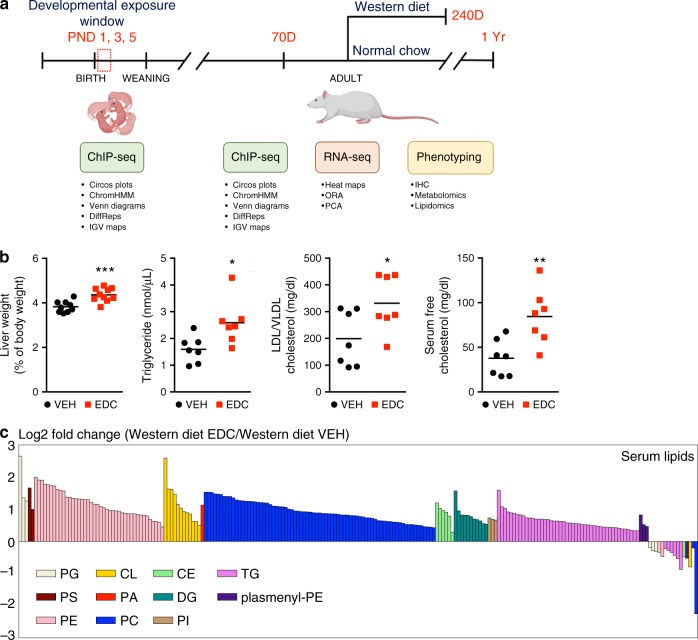
EDC-induced metabolic reprogramming in the setting of a Western-style diet. **a** Schematic of the integrated longitudinal epigenomic, transcriptomic and metabolomic analyses performed across the life-course in a rat model of early-life (postnatal days (PND)1–5) exposure to the EDC bisphenol A. Techniques and analytic approaches utilized at each life-stage are shown. **b** Metabolic phenotyping of vehicle- and EDC-exposed animals fed a Western-style diet at D240. Black circles represent individual vehicle (VEH) and red squares represent individual EDC (EDC) animals. *N* = 7 biologically independent animals per treatment. The *p* values generated by *t* test are indicated. *p < 0.05; **p < 0.01; ***p < 0.001. **c** EDC-exposure causes dyslipidemia in exposed animals fed a Western-style diet as seen in the targeted serum lipidomic analysis of vehicle- and EDC-exposed animals (*N* = 5 biologically independent animals per treatment). This analysis identified phospholipid [phosphatidylglycerol (PG), phosphatidylserine (PS), phosphatidylethanolamine (PE), phosphatidic acid (PA), phosphatidylcholine (PC), and phosphatidylinositol (PI)], cardiolipin (CL), cholesteryl ester (CE), diacylglycerol (DG), triglyceride (TG), and plasmenyl-PE levels as significantly (*q* < 0.25) increased in EDC-exposed animals on Western-style diet compared to VEH-exposed animals fed the same diet. Each bar is a different specific lipid within a color-coded class for each lipid. Source data for **b**, **c** are provided as a Source data file.

Early postnatal life is a critical window for murine liver development^20,21^, and developmental exposure to several EDCs has been shown to cause adult metabolic disease in animal models^9–11,15^. To elucidate the mechanism(s) by which early-life environmental exposure could cause metabolic dysfunction in adulthood, integrated longitudinal epigenomic, transcriptomic and metabolomic analyses were performed in Sprague-Dawley rats after a brief neonatal exposure to BPA (Fig. 1a). The low dose (50 μg/kg body weight) and brief exposure window (postnatal day (PND) 1, 3, and 5) produced no observable gross change in liver histology in young adult animals and had a minimal phenotype impact: EDC-exposed animals at D70 exhibited no increase in body weight, had significantly lower serum triglycerides, showed no net change in serum or liver lipids relative to vehicle controls (Supplementary Fig. 1a–d).

Diet composition is a known risk factor for metabolic disease in humans^22,23^ and a Western-style diet (high in fat, fructose, and cholesterol) has been used to promote metabolic dysfunction in animal models. Although EDC-exposed animals exhibited no overt metabolic dysfunction at D70, when subsequently placed on a Western-style diet (Fig. 1a), male, but not female, rats exhibited an altered metabolic phenotype that distinguished them from vehicle (VEH)-exposed counterparts on the same diet (see Supplementary Fig. 1f and Methods for information on the sex-bias for this phenotype). Adult male rats exposed to EDC as neonates exhibited significantly enlarged livers but no change in body weight or increase in serum alanine aminotransferase (ALT) levels, a measurement of liver damage (Fig. 1b and Supplementary Fig. 1e). They also exhibited an increase in serum triglycerides and developed dyslipidemia, with significantly increased serum LDL/VLDL accompanied by increased serum cholesterol (Fig. 1b) relative to age-matched VEH-exposed animals on the same diet. Unbiased lipidomic analysis identified phospholipid [phosphatidylglycerol (PG), phosphatidylserine (PS), phosphatidylethanolamine (PE), phosphatidic acid (PA), phosphatidylcholine (PC), and phosphatidylinositol (PI)], cardiolipin (CL), cholesteryl ester (CE), diacylglycerol (DG), triglyceride (TG), and plasmenyl-PE levels as significantly increased in EDC-exposed animals on Western-style diet compared to VEH-exposed animals on this diet (Fig. 1c). This phenotype was not caused by global organ-level metabolic disruption, as no significant differences were observed in bile acids levels (0.5868 versus 0.5276 μmol/g liver tissue), glucose (197.6 versus 195.3 mg/dl), plasma insulin (3.3 versus 3.1 ng/ml) or circulating FGF21 (1277.1 vs. 1236.7 pg/ml) in VEH- versus EDC-exposed animals fed a Western diet, respectively.

### Neonatal EDC-exposure reprograms the epigenome

To understand how a neonatal EDC exposure could reprogram adult metabolism, we focused on the liver, which plays a central role in whole-body metabolism. In neonatal and adult liver, chromatin immunoprecipitation sequencing (ChIP-seq) was performed for epigenetic histone modifications used to globally define epigenomic states^24–26^. These were histone 3, lysine 4, mono- and tri-methyl (H3K4me1 and H3K4me3, respectively), two activating histone marks found at enhancers and promoters; as well as histone 3, lysine 27 trimethyl (H3K27me3), a facultative repressive mark, and its cognate histone 3, lysine 27 acetyl (H3K27ac) mark, which also marks active promoters and enhancers.

ChIP-seq analyses were conducted to identify epigenomic changes that were: (1) associated with normal maturation of the liver between neonatal (PND5) and adult (D70) animals; (2) directly altered by EDC exposure in the neonatal liver (PND5 EDC- versus VEH-exposure); and (3) significantly different between adult (D70) EDC-reprogrammed and VEH-exposed liver. Focusing this analysis on neonatal and young adult animals prior to liver metabolic dysfunction onset allowed us to identify EDC-induced epigenetic changes that preceded, and could potentially direct, changes in gene transcription and metabolic function, versus changes occurring in response to diet and altered metabolism. For each active and repressive histone modification, differential peaks were identified using DiffReps, with significance achieved at *q* < 0.01 and fold change exceeding 2×. Venn diagrams were created for histone modifications associated with target genes [differential peaks within (±3) kb of the transcriptional start site (TSS)] or enhancers (compiled by the Fantom5 consortium^27,28^).

**Fig. 2:**
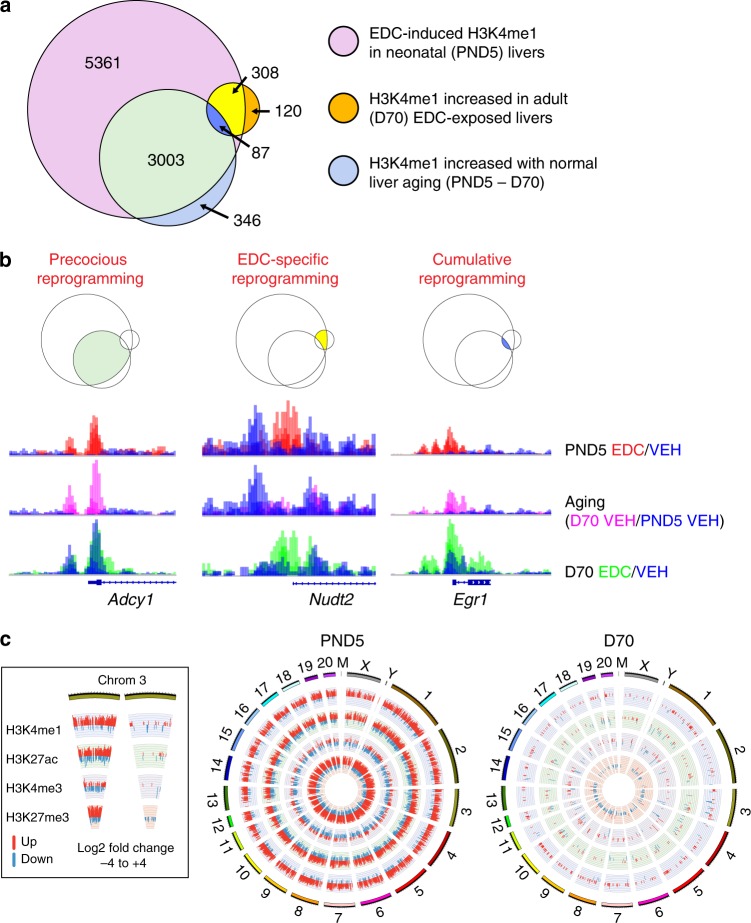
EDC exposure hijacks age-related epigenomic plasticity to accelerate epigenomic aging. **a** Venn diagrams for increased H3K4me1 focused on histone modifications associated with target genes, defined as having differential peak calls within ±3 kb of the transcriptional start site (TSS), using the GENCODE gene model and the BEDTOOLS software (*N* = 3 biologically independent animals per timepoint/treatment). **b** Integrated genome viewer (IGV) showing representative examples of each type of reprogramming for increased H3K4me1 at target genes: Precocious Reprogramming illustrated by *Adcy1*, EDC-specific Reprogramming illustrated by *Nudt2, and* Cumulative Reprogramming illustrated by *Egr1*. The data shown on the *y*-axis are the ChIP-seq read counts normalized to 1 million mapped reads. **c** Circos plots of differential peaks identified by chromatin immunoprecipitation sequencing (ChIP-seq) at target genes for active and repressive epigenetic histone modifications. Epigenomic data were analyzed using the rn6 rat genome build and the latest GENCODE gene model. For each of the four histone modifications, differential peaks that distinguished EDC (*N* = 3 per timepoint) from vehicle (*N* = 3 biologically independent animals per timepoint) exposed neonates (PND5) and adults (D70) were identified using DiffReps, with significance achieved at *q* < 0.01 and fold change exceeding 2×. Peaks with enrichment of the histone mark are indicated in red, while peaks with loss of the histone mark are indicated in blue.

Initially, Venn diagrams identified genes where epigenomic changes occurred associated with age and/or EDC exposure. This is illustrated for H3K4me1 (Fig. 2a), and H3K4me3, H3K27me3, and H3K27ac in Supplementary Fig. 2. For this analysis, changes in histone marks associated with normal maturation of the liver between neonatal and adult life were first identified (labeled as PND5-D70), and compared to differences in histone marks between EDC- and vehicle-exposed livers seen immediately after EDC exposure (labeled as PND5) or in adult animals (labeled as D70). As illustrated for H3K4me1 (Fig. 2a), Venn diagrams identified genes affected by three classes of epigenetic reprogramming: Precocious Reprogramming where EDC exposure accelerated acquisition of an adult epigenomic signature (characterized by the acquisition of adult liver histone marks in the neonatal liver); EDC-specific Reprogramming where changes in histone marks were induced acutely and remained significantly different between adult EDC- and VEH-exposed animals, but did not occur during normal liver maturation; and Cumulative Reprogramming, where liver maturation changes were further exaggerated by EDC exposure (Fig. 2b).

Remarkably, EDC-exposure induced accelerated epigenomic aging at 3003/3436 genes (87%) where H3K4me1 normally increased with age during liver maturation (Fig. 2a). This Precocious Reprogramming is illustrated in Fig. 2b with *Adcy1*. In neonatal liver, EDC exposure induced an H3K4me1 peak at the *Adcy1* TSS, the same position where H3K4me1 increased with age during normal liver maturation. Consequently, the H3K4me1 peak was indistinguishable at D70 between EDC- and VEH-exposed animals (Fig. 2b). This is further illustrated with the Circos plots shown in Fig. 2c. EDC-exposure induced genome-wide epigenomic reprogramming of target genes in neonatal liver at PND5, with H3K4me1, H3K27ac, and H3K27me3 exhibiting more EDC-induced changes than H3K4me3. This finding mirrors, and is largely driven by, the Precocious Reprogramming shown in the Venn diagrams. Thus, at D70, far fewer epigenomic differences are evident when livers from EDC-exposed animals are compared to livers from VEH-exposed animals, reflecting the acceleration of normal epigenomic aging by EDC exposure (Fig. 2c).

Additional examples of Precocious Reprogramming (*Kcnk15, Fzd2, Prex1*, and *Rims1)* are shown in Supplementary Fig. 2b. Altogether, EDC exposure accelerated epigenetic aging and induced an adult H3K4me1 signature at 3090 [3003 Precocious + 87 Cumulative (see below)] genes. Interestingly, H3K4me1 decreased with age at far fewer genes (475 as shown in Supplementary Fig. 2a), and the impact of EDC-induced reprogramming at these genes was minimal: only 64/475 genes (14%) exhibited precocious decreases in H3K4me1. This suggests that age-associated changes in H3K4me1 were not a reflection of generalized tissue aging.

EDC-exposure also accelerated epigenetic aging of H3K27ac and H3K27me3. As shown in Supplementary Fig. 2a, at 5575/6783 genes (82%) where H3K27ac increased with age and 909/1830 genes (50%) where this mark decreased with age, EDC exposure induced Precocious Reprogramming and accelerated epigenetic aging, with neonatal livers acquiring an adult H3K27ac signature at PND5. For H3K27me3, precocious increases in this mark occurred at 4666/6127 genes (76%) and decreases at 513/1492 genes (34%) where this mark normally changed with age.

Accelerated epigenomic aging was not observed for all marks, and specificity was observed even within targets for a given epigenetic writer. The COMPASS complex is responsible for both the H3K4me1 and H3K4me3 histone methyl marks^29^, with the histone methyltransferase MLL3/4 writing the H3K4me1 and other SET/MLL methyltransferases writing the H3K4me3 mark. In contrast to observations for H3K4me1, the primary H3K4me3 change with age was a decrease rather than increase: 363 genes exhibited increases whereas 861 genes exhibited decreases in H3K4me3 during normal liver maturation, an order of magnitude less than was observed for other marks. In contrast to other histone marks, EDC exposure had little effect on age-related increases in H3K4me3, with only 55/363 (15%) exhibiting Precocious Reprogramming, whereas EDC-exposure decreased H3K4me3 at 338/861 (39%) genes where this change normally occurred with age (Supplementary Fig. 2). Overall, the 393 genes with Precocious Reprogramming of H3K4me3 were fewer than seen for H3K4me1, H3K27ac, and H3K27me3, histone marks that retained greater epigenomic plasticity during liver maturation.

While accelerated epigenetic aging accounted for >98% of the observed developmental reprogramming, a subset of genes exhibited EDC-specific or Cumulative Reprogramming, primarily due to changes in H3K4me1. EDC-specific Reprogramming of H3K4me1 occurred in 308 genes, as illustrated by *Nudt2* (Fig. 2b), *Hox10d and Zbtb4* (Supplementary Fig. 2b*)*. *Nudt2* showed no H3K4me1 increase with age, but EDC exposure increased H3K4me1 at TSS at PND5, and this reprogramming persisted into adulthood (D70). Cumulative Reprogramming of H3K4me1 occurred at 87 genes, including an important regulator of liver metabolism, early growth response 1 (*Egr1)*. For *Egr1*, the age-related H3K4me1 increase seen at the TSS was further exaggerated by EDC exposure, and persisted in EDC- versus VEH-exposed animals at D70 (Fig. 2b). Cumulative Reprogramming examples for other genes (*Fam181b* and *Cpm)* are shown in Supplementary Fig. 2b. Finally, reprogramming was seen in D70 livers of EDC-exposed animals that was not directly induced by EDC exposure (i.e. not observed at PND5) nor associated with normal epigenomic aging of the liver. However, these later-onset changes in the livers of EDC-exposed animals comprised <2% of all reprogramming observed (Fig. 2a and Supplementary Fig. 2a).

Enhancer elements also exhibited Precocious Reprogramming of H3K4me1 and H3K27ac, which mark this important class of *cis*-acting transcriptional regulators^30^. A lift-over of the comprehensive mouse Fantom5 enhancer data identified 25,527 corresponding rat enhancer elements. The vast majority of EDC-induced changes at these enhancers were Precocious Reprogramming. 877/1522 enhancers (58%) that gained/lost H3K4me1 during normal epigenetic aging exhibited Precocious Reprogramming, as did 2551/4530 enhancers (56%) that gained/lost H3K27ac (Supplementary Fig. 2c), demonstrating that in addition to promoters, early-life EDC exposure accelerated epigenome aging of enhancers as well.

We next asked which chromatin states were the targets of, and most vulnerable to, developmental reprogramming by EDC exposure (Supplementary Fig. 3A). ChromHMM provides an assessment of combinatorial and/or spatial changes in histone modifications, and was used by both Encode and NIH Epigenome Roadmap consortia to integrate multiple histone modifications data using a Hidden Markov Model^24–26^. For each mark and epigenomic state, we determined differential peak overlaps and the odds-ratio enrichment based on the total epigenomic state basepair size relative to the genome size.

This analysis revealed that chromatin states associated with age-related increases in H3K4me1, H3K27ac, and H3K27me3 in liver were the primary reprogramming targets, defined by an odds-ratio enrichment ≥ 100. H3K4me1, H3K27ac and H3K27me3 marks were enriched at Flanking Bivalent TSS/Enhancers, Active TSS, Bivalent/Poised TSS and Bivalent Promoters (Supplementary Fig. 3b). These epigenomic states retained the most epigenomic plasticity in neonatal liver, defined as having the greatest odds-ratio enrichment between neonatal (PND5) and adult (D70) liver. In contrast, H3K4me3, which exhibited little increase with age, exhibited virtually no EDC-induced enrichment at any chromatin state, even at Bivalent Promoters where this mark normally increased with age. Interestingly, while there was no depletion of H3K4me1, H3K27ac and H3K27me3 for any epigenomic state, decreased H3K4me3 occurred at Bivalent TSS/Enhancers, the same epigenomic state where this mark decreases during normal liver aging (Supplementary Fig. 3c).

### Neonatal EDC exposure reprograms metabolic gene expression

**Fig. 3:**
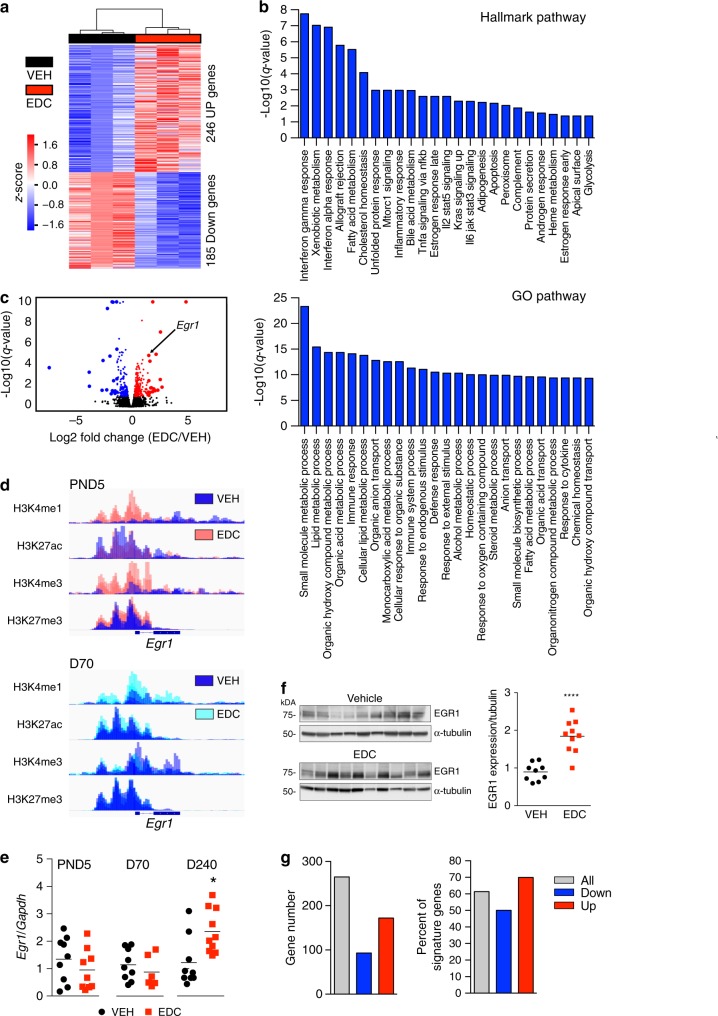
Transcriptional profiling reveals reprogramming of the EGR1 transcriptome. **a** Transcriptomic profiling by RNA-seq of vehicle- (VEH black bar) and EDC- (EDC red bar) exposed livers animals fed a Western-style diet. *N* = 3 biologically independent animals per treatment. Heatmap showing DEseq differential gene analysis, with significance achieved at *q*-value < 0.1 and fold change exceeding 1.25×. **b** Overrepresentation analysis (ORA) of differentially expressed genes performed using a hypergeometric test, with significance achieved at *q* < 0.2, against the Hallmark gene set compendium and the GO database. **c** Volcano plot of the transcriptomic footprint from EDC-exposed vs VEH-exposed livers of animals fed a Western-style diet. Red circles indicate upregulated, while blue circles indicate downregulated genes, respectively. Larger circles identify the top 20 up- and downregulated genes. **d** Reprogramming of histone marks for *Egr1* illustrates Precocious Reprogramming of H3K4me1 and Cumulative Reprogramming of H3K27me3 shown with IGV browser plots using ChIP-seq data obtained at PND5 (upper) and D70 (lower). **e** RT-qPCR analysis of *Egr1* gene expression in the liver of animals at PND5, D70, or D240 after feeding a Western-style diet. Vehicle (VEH) = black dots, EDC = red dots. *N* = 9 biologically independent animals for VEH and *N* = 10 biologically independent animals for EDC. The *p* value generated by *t* test is indicated. *p < 0.05. **f** Western analysis for EGR1 expression in EDC- vs VEH-exposed livers (left) and quantitation (right). *N* = 9 biologically independent animals for VEH and *N* = 10 biologically independent animals for EDC. The *p* value generated by *t* test is indicated. ****p < 0.0001. **g** EGR1 targets within the significant differentially expressed gene signature (**a**) were identified using the Harmonizome database (‘All’ shown with gray bars). EGR1 targets are represented as total gene number (left) or as the percent of the differentially expressed genes (right). Genes that were increased are represented as ‘Up’, and genes that were decreased in the EDC signature are represented as ‘Down’. Source data for **a**, **b** and **e**–**g** are provided as a Source data file.

To explore how epigenetic reprogramming impacted gene expression, we employed transcriptional profiling by RNA-sequencing (RNA-seq), then executed DESeq2 differential gene analysis, with significance achieved at *q*-value <0.1 and fold change exceeding 1.25×. Hierarchical clustering identified 431 genes (246 up and 185 down) differentially expressed in EDC-reprogrammed animals on Western-style diet relative to age-matched VEH-exposed animals on the same diet (Fig. 3a). The top 20 differentially expressed genes (up and down) are shown in Supplementary Table 1. Principal component analysis (PCA) confirmed a robust differential response to Western-style diet in EDC-exposed animals (Supplementary Fig. 4a), with clear separation between EDC- and VEH-exposed groups. Genes were sorted by their contribution to the top three principal components, and the top 25 major effect genes associated with variance in each component are listed in Supplementary Table 2.

Overrepresentation analysis (ORA) using Hallmark and GO databases (Fig. 3b) revealed enrichment for metabolism-associated pathways in EDC-reprogrammed livers of animals fed a Western-style diet. The top 25 Hallmark pathways included cholesterol, xenobiotic, and fatty acid metabolism. Enrichment was also seen for hormone response pathways, which was interesting given the function of this EDC as a nuclear hormone receptor ligand engaging estrogen receptor and related pathways^31,32^. Enriched GO pathways included lipid and sterol metabolism (Fig. 3b).

Although largely metabolically silent prior to feeding with Western Diet, and subsequent development of an overt liver phenotype, reprogramming of metabolism-linked gene expression was evident as early as D70, (Supplementary Fig. 4c and d, and Supplementary Table 3). Differential transcriptomic analyses using DESeq2 at D70 of EDC- versus VEH-exposed livers showed significant changes in 592 differentially expressed genes (278 up and 314 down); corresponding hierarchical clustering is shown in Supplementary Fig. 4b. ORA analysis using Hallmark and GO databases (Supplementary Fig. 4c and d), showed enrichment for fatty acid, xenobiotic, and lipid metabolism, as well as steroid hormone and steroid signaling. Of these 592 differentially expressed genes, 55% of genes with increased expression (153/278) and >60% of genes with decreased expression (194/314) were targets for Precocious Reprogramming (Supplementary Fig. 4e). In contrast, comparatively few transcriptional changes could be ascribed to EDC-specific or Cumulative Reprogramming: 21/592 (~4%) and 7/592 (~1%), respectively.

### EGR1 transcriptional response is epigenetically reprogrammed

As shown in the volcano plot in Fig. 3c, *Egr1* was one of the top genes overexpressed in liver of EDC-reprogrammed animals on Western-style diet. The EGR1 transcription factor responds to diet and stress, and controls genes involved in liver metabolism^33^. As shown in Fig. 3d, *Egr1* was a target for H3K4me1 Cumulative Reprogramming and H3K27me3 Precocious Reprogramming. Notably, the impact of this epigenomic reprogramming was silent (i.e., no change in *Egr1* expression by RNA-seq or RT-PCR) in EDC-reprogrammed neonatal (D5) and adult (D70) livers, indicating that reprogramming of these histone marks was not a consequence of increased *Egr1* expression (Fig. 3e). However, once reprogrammed animals were fed a Western diet, *Egr1* expression (Fig. 3e) and protein levels (Fig. 3f) were significantly increased compared to controls on the same diet.

To determine if increased EGR1 expression led to increased transcription of downstream targets, the Harmonizome database^34^ was used to identify EGR1 targets in the expression signature of reprogrammed animals fed a Western-style diet. This analysis revealed that among genes differentially expressed in livers of animals on Western-style diet, >60% were EGR1 targets, including both up- and downregulated genes (Fig. 3g). In addition to epigenetic reprogramming of the *Egr1* gene itself (Fig. 3d), of the 431 genes differentially expressed in the setting of Western-style diet, 206 were targets for Precocious Reprogramming and 158 of these (77%; 158/206) were EGR1 targets (Supplementary Fig. 4f). Similar to our observations at D70, very few D240 differentially expressed genes were Cumulative or EDC-specific Reprogramming targets, as only 4 and 5 reprogrammed genes, respectively, were differentially expressed.

### Epigenome:environment interactions impact liver metabolism

**Fig. 4:**
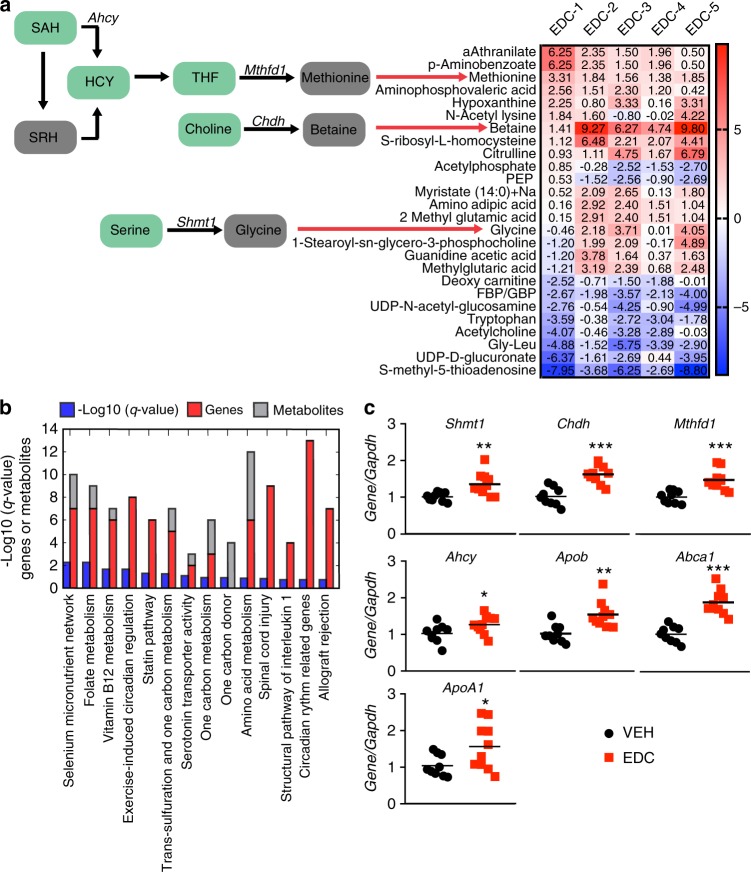
Reprogramming by early-life EDC exposures causes metabolic disruption in the liver. **a** Targeted metabolomics of livers of animals fed a Western-style diet. For each metabolite and each EDC-exposed animal, we computed its z-score (number of standard deviations) compared to the vehicle-treated animals. *N* = 5 biologically independent animals per treatment. The results were visualized with a CBioPortal Oncoprint-type exploration tool, and metabolites increased by at least 1.5 standard deviations in at least 3 animals and down in none or decreased by at least 1.5 standard deviations in at least 3 animals and up in none are shown. Genes that encode enzymes associated with altered metabolites are depicted on the left in italics (*Ahcy*, *Mthfd1*, *Chdh*, and *Shmt1*). S-Adenosylhomocysteine = SAH, homocysteine = HCY, S-ribosyl homocysteine =  SRH, tetrahydrofolate = THF, phosphoenolpyruvic acid = PEP, fructose-bisphosphate/glucose-bisphosphate = FBP/GBP, glycine = Gly, leucine = Leu. **b** Metabolism set enrichment analysis (MetSEA) using genes identified by RNA-seq (shown in Fig. 3a) and metabolites identified in (**a**) to elucidate metabolic pathways disrupted in EDC-reprogrammed liver. MetSEA was performed using integrated recurrently up- or downregulated metabolites from livers of EDC-exposed animals with transcriptional signatures using the hybrid metabolite/transcriptomics pathway compendium WikiPathways. ORA using a hypergeometric distribution against WikiPathways identified 14 significantly enriched pathways at *q* < 0.2. **c** RT-qPCR validation of differentially expressed genes in key metabolic pathways identified by RNA-seq and MetSEA. Data from an independent validation set of vehicle-exposed (VEH; black dots) and EDC-exposed (EDC; red squares) animals fed a Western-style diet are shown. *N* = 9 biologically independent animals for VEH and *N* = 10 biologically independent animals for EDC. The *p* values generated by *t* test are indicated. *p ≤ 0.05; **p < 0.01; ***p < 0.001. Source data for **c** are provided as a Source data file.

To link reprogramming of the EGR1 transcriptome to altered liver metabolism, we performed targeted metabolomics on livers of animals fed a Western-style diet. We used a CBioPortal platform^35^ approach to generate an Oncoprint-type heatmap, which identified 16 recurrently increased and 10 recurrently decreased metabolites in livers of EDC-exposed animals (Fig. 4a). We next integrated recurrently up- or downregulated metabolites from EDC-exposed animals with transcriptional signatures using the hybrid metabolite/transcriptomics pathway compendium WikiPathways^36^. We performed a metabolomics set enrichment analysis (MetSEA) by combining metabolomic and RNA-seq data (Fig. 4b and Supplementary Table 4). This approach identified alterations in one-carbon metabolic pathways (5 of the top 10 pathways) as enriched in EDC-reprogrammed animals fed a Western-style diet.

Driving the one-carbon metabolism enrichment were increases in the metabolites glycine, betaine and methionine (Fig. 4a and Supplementary Table 4) and increased expression of genes involved in their metabolism: *Chdh* (choline dehydrogenase), *Shmt1* (serine hydroxymethyltransferase 1), *Mthfd1* (methylenetetrahydrofolate dehydrogenase 1), and *Ahcy* (S-adenosylhomocysteine hydrolase). To validate the aberrant expression of these genes in reprogrammed animals, we confirmed altered expression of *Chdh, Shmt1, Mthfd1*, and *Ahcy* in livers by RT-PCR in a validation set of adult animals fed a Western-style diet with prior exposure to EDC as neonates (*N* = 7) relative to vehicle controls (*N* = 9) on the same diet (Fig. 4c). These data confirmed significantly increased expression of *Chdh* (*p* < 0.0001), *Shmt1* (*p* < 0.0058), *Mthfd1* (*p* < 0.0004) and *Ahcy* (*p* < 0.0490) in livers of EDC-exposed animals. Additional genes involved in one-carbon, lipid and/or cholesterol metabolism were confirmed by RT-PCR in the test and validation cohorts (Fig. 4c): *Apob*, encoding apolipoprotein B, a building block critical to formation of very low-density lipoprotein (VLDL) (*p* < 0.0018), *Abca1*, encoding the ATP-binding cassette transporter named cholesterol efflux regulatory protein (CERP) (*p* < 0.0001), and *ApoA1*, encoding apolipoprotein A-1, a component of high-density lipoprotein (HDL) (*p* < 0.0358).

Finally, the conditional nature of EDC-induced metabolic dysfunction, and its dependence on later-life diet, was shown by examining expression of reprogrammed genes in EDC- versus VEH-exposed animals at one year of age fed normal chow. No significant differences were seen by RT-PCR in expression of *Shmt*, *Chdh*, *Mthfd1*, *Ahcy*, *Apob, Abca1, ApoA1*, or any of 19 reprogrammed genes examined in these animals (Supplementary Fig. 5a). The key role of diet in distinguishing between reprogrammed and non-reprogrammed animals was further confirmed by PCA analysis of the transcriptional profiles of D70 EDC- versus VEH-exposed liver using the same principal components that separated EDC- from VEH-exposed animals fed a Western diet at D240. Principal components separating EDC-reprogrammed animals from vehicle controls on Western-style diet at D240 failed to separate EDC- versus VEH-exposed livers of D70 animals (Supplementary Fig. 5b), highlighting the critical interaction between early-life reprogramming and later life diet.

**Fig. 5:**
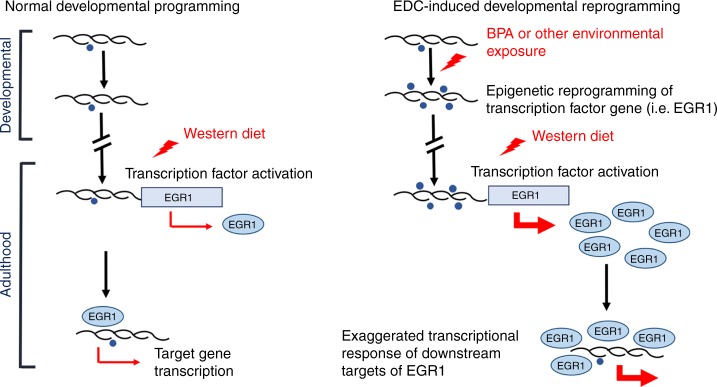
Model for developmental reprogramming of *Egr1* by early-life EDC exposure. Schematic illustrating programming of Egr1 during normal development (left) and reprogramming of Egr1 by early-life EDC exposure (right). Reprogramming may remain transcriptionally and phenotypically silent until triggered by a later life event, such as a dietary challenge.

Together, these data suggest a model for how both early and late epigenome:environment interactions can potentially impact liver metabolic function. In this model (Fig. 5), early-life epigenome:environment interactions can reprogram the developing epigenome, and cause life-long changes in histone modifications at key target genes and epigenomic states. This epigenomic reprogramming may remain transcriptionally and phenotypically silent until triggered by a later life event, such as a dietary challenge. In the case of the EDC BPA, *Egr1* reprogramming and diet-induced increased expression resulted in altered gene expression and production of metabolites in the cholesterol, lipid and one-carbon metabolic pathways that drove metabolic dysfunction in adulthood. Thus, both early- and later-life epigenome:environment interactions play important roles in regulating hepatic metabolism across the life-course.

## Discussion

We demonstrated that early-life EDC exposure can reprogram the liver epigenome to create a conditional vulnerability to diet-triggered adulthood metabolic dysfunction. Mechanistically, developmental EDC exposure induced premature epigenomic aging in H3K4me1, H3K27ac, and H3K27me3 epigenetic marks in the liver, which targeted genes and chromatin states with age-related plasticity, and mimicked changes normally occurring during liver maturation with age. These alterations were observed with short-term exposure (on neonatal days 1, 3, and 5) to the chronic oral Reference Dose (by definition, likely to be without lifetime risk of deleterious effects) for BPA of 50 μg/kg body weight/day (USEPA, 2017. IRIS (Integrated Risk Information System). Environmental Protection Agency, Washington, DC. http://www.epa.gov/iris/index.html).

Our data add to the growing awareness that epigenome:environment interactions play important roles in health and disease susceptibility both early and late in life. During early life, the epigenome is plastic, undergoing remodeling as part of normal development and aging processes. This plasticity creates a vulnerability to environmental exposures, which can disrupt the epigenome, and for in the case of the EDC BPA, accelerate normal epigenomic aging to cause widespread epigenetic reprogramming. Later in life, epigenome:environment interactions can unmask the impact of this reprogramming, with the reprogrammed epigenome exhibiting aberrant responses to environmental challenges (ex. a Western-style diet). The emerging paradigm of early-life reprogramming by, and altered later-life response to, the environment is a central theme of DOHaD research, and has been observed in organs other than the liver^37,38^. We have shown in prostates reprogrammed by early-life EDC exposure that an adult testosterone challenge leads to exaggerated expression of reprogrammed androgen-responsive genes^37^, and in the uterus, reprogramming causes estrogen-responsive genes to become hyper-responsive to even low estrogen levels^38^. However, our unbiased metabolomic data also hint that not all EDC-induced reprogramming effects may adversely affect health: BPA-exposed animals on normal diet exhibited decreased serum triglycerides and ALT activity at D70 relative to vehicle-exposed controls (Supplementary Fig. 1b and e).

Currently, little is known about how early-life exposures affect epigenomic aging. Our data reveal EDC-exposure can induce accelerated aging of histone marks, which exhibit modifier-, mark- and gene-specificity. For active marks, H3K4me1 and H3K27ac were preferentially altered by EDC exposure relative to H3K4me3, which correlated with the amount of age-related plasticity associated with these histone marks in the neonatal liver. We speculate that retained plasticity may reflect the function of these histone marks. For example, addition of H3K4me3 at promoters of liver-specific genes may occur primarily during organogenesis (a developmental window that precedes our window of exposure), reducing plasticity of this epigenetic mark in the neonatal liver. Other amplifying (H3K4me1 and H3K27ac) or dampening (H3K27me3) histone marks may remain more plastic until liver maturation completes, and thus be more vulnerable to perturbation by environmental exposures during early life (and our exposure window), when the liver is undergoing functional maturation^20,21^. Another implication of our data is that studies to determine impact of developmental reprogramming by assessing the adult epigenome could miss reprogramming events that accelerate normal epigenetic aging: in adult livers, genes that were targets for Precocious Reprogramming were indistinguishable between exposed and unexposed livers. For genes targeted by Precocious Reprogramming, the impact of early-life EDC exposure was only apparent when epigenetic signatures were compared early between exposed and unexposed neonates.

To date, the vast majority of epigenome:environment studies using the EDC BPA have focused on DNA methylation^9,11,39–44^. Of these, only a handful assessed the transcriptional impact of altered DNA methylation and functional outcomes in the liver^40,43^. Only one study has examined perinatal BPA exposure effects on liver histone modifications, and the effect of these modifications on gene expression and liver function were not reported^44^. In contrast to the little that is known regarding the impact of epigenome:environment interactions on histone modifications, aging-associated changes in DNA methylation (DNAm-aging) have become well-established since the original studies of Horvath^45^, and reflect the impact of both environmental exposures and pathophysiological processes^41,46–51^. Previous studies have assessed epigenetic signatures in rodent and human liver during normal aging by assessing DNAm-aging^52–55^. In liver, DNAm-aging can be accelerated under conditions such as obesity^56,57^, in utero malnutrition^58^, ovariectomy^59^, and alcohol dependence^48^, and is seen in patients with nonalcoholic steatohepatitis^60^. Accelerated epigenetic aging of histone modifications by early life chemical exposure opens new avenues for exploring, and understanding, how early life exposures impact health trajectory across the life course.

In our study, we observed changes in both active marks (H3K4me1, H3K4me3, and H3K27ac) and repressive marks (H3K27me3), and reprogrammed genes exhibited both increased and decreased transcription. One pathway by which early-life exposures reprogram the epigenome is by altering the activity of epigenetic programmers that add and/or remove histone methyl marks via several different mechanisms^12^. An early-life exposure that increases or decreases the activity of epigenetic programmers will cause a concomitant increase or decrease in their cognate methyl mark in the reprogrammed epigenome. Thus, if an exposure affects a histone methyltransferase/demethylase for an active mark and a methyltransferase/demethylase for repressive mark, it would not be surprising to see reprogramming of both active and repressive marks. Similarly, the transcription impact when a specific mark is gained or lost will depend on the nature of the mark itself. For example, gain of an active mark or loss of a repressive mark could both increase gene expression.

Our findings in this longitudinal investigation into the early-life EDC exposure impact overlap with human epidemiological studies reporting higher urinary levels of BPA are associated with adverse liver changes in young children^61,62^ and with increased risk of NAFLD in adolescents^63^. One caveat of our study is that non-fasted insulin levels, insulin sensitivity, and glucose tolerance were not measured. Adding these measurements in future studies would provide a picture of systemic alterations that may synergize with EDC-mediated liver reprogramming to adversely affect health. While a comprehensive mechanistic understanding of the association of BPA exposure with hepatic disturbances in humans remains incompletely understood, our interrogation of the rodent hepatic epigenomic and transcriptional effects of an early-life environmental exposure provides insights for understanding how exposure to EDCs, such as BPA, may alter epigenetic states leading to increased metabolic disease risk.

While these findings support the concept that the epigenome influences the pathogenesis of metabolic dysfunction, it is clear that overnutrition is a key driver of the current epidemic of metabolic disease. Indeed, our finding that animals taken to one year after exposure on a normal diet did not exhibit the changes in metabolism-linked gene expression seen in those fed a Western diet corresponds with this fundamental concept, and with past observations that dietary manipulation can slow or reverse epigenetic changes associated with aging in the liver^64–67^. Our findings raise the possibility that early life environmental exposure to EDCs or other chemicals may be an overlooked risk factor increasing risk of developing metabolic dysfunction by altering the response to diets high in fat, fructose and cholesterol.

An improved understanding of how early-life environmental exposures may increase metabolic disease risk, possibly through accelerated epigenetic aging, has the potential to guide the development of innovative strategies to prevent and manage adult metabolic disease. The conditional nature of liver metabolic perturbation caused by EDC exposure implies that it may be possible to develop interventions to prevent, ameliorate, or reverse effects of early-life environmental exposures that increase risk of metabolic disease later in life. This possibility is particularly relevant for children and adolescents, who exhibit a rapidly increasing prevalence of metabolic disease^68^. Thus, our data provide a springboard for DOHaD efforts to understand environmental factors contributing to the current NAFLD epidemic^10,18,19^.

Also of note, while we focused on liver due to its role as governor of the entire organism’s metabolic state, environmental EDC exposures are usually systemic, making it likely that there were effects on other organs/tissues, which could also potentially impact liver function. For example, developmental exposure to low-dose BPA has been shown to increase pancreatic β-cell mass and induce hyperinsulinemia in non-fasted offspring later in life^69^, and excess insulin could also contribute to altered liver metabolism and subsequent liver disease. Coordinated efforts to characterize epigenomic reprogramming across many organs and target tissues, such as the liver, and more accessible surrogate tissues, including blood and skin, are currently being undertaken by the NIEHS TaRGET II Consortium^70^. The TaRGET II goal is to understand how several environmental toxicants, including BPA, reprogram the epigenome and to develop epigenetic biomarkers for early-life toxicant exposures that increase future disease risk. Findings of integrated longitudinal studies, such as we present here, will inform TaRGET II and similar initiatives aimed at identifying signatures for epigenomic reprogramming by environmental exposures. It should also be noted that we observed a more robust response in males than in females exposed to BPA and fed the same diet (Supplementary Fig. 1f). Sex-specific modifications of gene expression and epigenome after developmental BPA exposure have been previously reported in rodent liver^44^, highlighting a need to examine potential sex-bias of observed phenotypes in future epidemiological studies. Our discovery that early-life EDC-exposure causes a pattern of epigenomic aging associated with greater risk of metabolic dysfunction in adult liver represents an early advance towards this goal.

## Methods

### Animal studies

Experimental procedures complied with all relevant ethical regulations for animal testing and research. These procedures were approved by the Institutional Animal Care and Use Committee at Texas A&M Institute of Biosciences & Technology. Sprague-Dawley rats aged 6–8 weeks were purchased from Harlan and used as breeders to produce rats for this study. One breeder pair was housed per cage. To reduce exposure to exogenous xenoestrogens, rats were housed in polycarbonate-free caging and were fed a phytoestrogen-reduced diet (Phytoestrogen Reduced II 18-5, Ziegler Bros, Inc) ad libitum. Cages and bedding were changed once per week. Rats were maintained on a 14-h light, 10-h dark cycle.

Neonatal rats were treated with vehicle (VEH; sesame oil) or bisphenol A (BPA; 50 µg/kg dissolved in sesame oil) orally via pipette tip on post-natal days 1, 3, and 5. Littermates of the same sex were randomly assigned to the treatment groups. BPA was obtained from the National Institute of Environmental Health Sciences (NIEHS). The dose and route of administration recapitulates human exposure to BPA. At day 180 (D180), adult rats in both treatment groups were fed a diet high in fat (40% kcal), fructose (20% kcal) and cholesterol (2%) (Western-style diet) for 60 days (D09100301, Research Diets, Inc). Rats were fasted overnight prior to tissue collection.

Liver tissue was harvested on post-natal day 5 at 6 h after treatment (PND5; VEH: *n* = 59 (34 males and 25 females); BPA: *n* = 37 (22 males and 15 females)), on day 70 (D70; VEH: *n* = 37 (19 males and 18 females); BPA: *n* = 30 (15 males and 15 females)), and on day 240 after challenge with Western-style diet (D240; VEH: *n* = 20 (9 males and 11 females; BPA: *n* = 17 (10 males and 7 females)). Tissue was snap-frozen in liquid nitrogen for downstream transcriptomic, epigenomic, and metabolomic analyses or fixed in 10% neutral buffered formalin and stored in 70% ethanol before processing and paraffin embedding by the Research Histology, Pathology and Imaging Core at The University of Texas MD Anderson Cancer Center, Science Park. Additionally, blood was collected from D70 and D240 animals via cardiac puncture at the time of tissue harvest. For separating serum from the blood cells, the samples were allowed to clot at room temperature for 20–30 min, followed by centrifugation for 10 min at 1000 × *g*, and storage of the separated serum at −80 °C. Body weight was recorded at the time of tissue harvest for D70 and D240 rats. Liver weight was recorded at the time of tissue harvest for D240 rats.

Female rats failed to show the same robust response to EDC-exposure seen in males, and for the purposes of the present study, we focused our analysis on male rats. This assessment was made based on key metabolic, gene expression and epigenomic indicators. Female rats on Western-style diet failed to exhibit the increase in serum triglycerides seen in the males: vehicle (*N* = 6) and EDC (*N* = 7) exposed female rats showed no significant difference in serum triglycerides, which were 0.9 nmol/ul and 1.7 nmol/μl, respectively *p* = 0.23. RT-PCR for genes that were overexpressed in the livers of male EDC-exposed animals on Western-style diet (*Shp*, *Ccne1*, and *Lrat*) showed no differences in expression between vehicle- and EDC-exposed females (Supplementary Fig. 1f). In males (D70), the promoter region of these genes exhibited an increase in H3K4me3 that correlated with elevated expression, but ChIP in female rat livers showed no increase for this epigenetic histone mark (Supplementary Fig. 1f).

### Serum alanine aminotransferase (ALT) assay

Serum ALT levels were determined in D240 (*n* = 7 per treatment group) male rats using Abcam’s Alanine Transaminase Activity Assay kit (ab105134) per the manufacturer’s instructions.

### Liver and serum lipidomics

Serum triglyceride levels were determined in D70 (*n* = 4 per treatment group) and D240 (*n* = 7 per treatment group) male rats using Abcam’s triglyceride quantification kit (ab65336) per the manufacturer’s instructions. Serum LDL/VLDL, total cholesterol, and free cholesterol levels were measured in D240 male rats (*n* = 7 per treatment group) using Abcam’s HDL/LDL/VLDL Cholesterol Assay kit (ab65390) per the manufacturer’s instructions.

For lipidomic analysis, frozen liver tissue and serum from D70 (*n* = 5 per treatment group) and D240 (*n* = 5 per treatment group) male rats were sent to the Metabolomics Core at Baylor College of Medicine for processing. Lipids were extracted using a modified Bligh–Dyer method. Fifty µL of serum and 25 mg of the crushed liver was used for the extraction. The extraction was carried out using 2:2:2 volume ratio of water/methanol/dichloromethane at room temperature after spiking internal standards 17:0 LPC, 17:0 PC, 17:0 PE, 17:0 PG, 17:0 ceramide, 17:0 SM, 17:0PS, 17:0PA, 17:0 TAG, 17:0 MAG, 16:0/18:1 DAG, 17:0 CE. The organic layer was collected and completely dried under nitrogen. Before MS analysis, the dried extract was resuspended in 100 μL of Buffer B (10:5:85 acetonitrile/water/Isopropyl alcohol) containing 10 mM NH_4_OAc and subjected to LC/MS. The lipidome was separated using reverse-phase chromatography.

For internal standards and quality controls, we used high-performance liquid chromatography (LC) grade acetonitrile, and dichloromethane from Sigma (St. Louis, MO), isopropanol from optima- liquid chromatography/mass spectrometry (LC/MS) Fisher (New Jersey, NJ), and methanol from J.T. Baker (Radnor, PA). We obtained water from a Millipore high purity water dispenser (Billerica, MA). We purchased MS grade lipid standards from Avanti Polar Lipids (Alabaster, AL). We prepared the lipid stock solution by weighing an exact amount of the lipid internal standards in chloroform/methanol/H_2_O resulting in a concentration of 1 mg/mL and stored at −20 °C. We further diluted the stock solutions to 100 pmol/μL by mixing appropriate volume of the internal standards LPC 17:0/0:0, PG 17:0/17:0, PE 17:0/17:0, PC 17:0/17:0, TAG 17:0/17:0/17:0, SM 18:1/17:0, MAG 17:0, DAG 16:0/18:1, CE 17:0, ceramide d 18:1/17:0, PA 17:0, PI 17:0/20:4, and PS 17:0/17:0. We used two kinds of controls to monitor the sample preparation and MS. To monitor instrument performance, we used 10 μL of a dried matrix-free mixture of the internal standards, reconstituted in 100 μL of buffer B (5% water, 85% isopropanol: 10% acetonitrile in 10 mM NH_4_OAc). To monitor the lipid extraction process, we used a standard pool of tissue samples made up of aliquots from these samples.

For data acquisition through LC/MS analysis, we used a Shimadzu CTO-20A Nexera X2 UHPLC system equipped with a degasser, binary pump, thermostatted auto sampler, and a column oven for chromatographic separation. The column heater temperature was set at 55 °C. For lipid separation, the 5 μL of the lipid extract was injected into a 1.8 μm particle 50 × 2.1 mm Acquity HSS UPLC T3 column (Waters, Milford, MA) which heats to 55 °C. Acetonitrile/water (40:60, v/v) with 10 mM ammonium acetate was solvent A and acetonitrile/water/isopropanol (10:5:85 v/v) with 10 mM ammonium acetate was solvent B. For chromatographic elution we used a linear gradient over a 20 min total run time, with 60% solvent A and 40% solvent B gradient in the first 10 min, then the gradient was ramped in a linear fashion to 100% solvent B which was maintained for 7 min. After that the system was switched back to 60% solvent B and 40% solvent A for 3 min. The flow rate used for these experiments was 0.4 mL/min and the injection volume was 5 μL. The column was equilibrated for 3 min before the next injection and run at a flow rate of 0.4 mL/min for a total run time of 20 min. The data acquisition of each sample was performed in both positive and negative ionization modes using a TripleTOF 5600 equipped with a Turbo V^TM^ ion source (AB Sciex, Concord, Canada). The column effluent was directed to the electrospray ionization source. The voltage of source was set to 5500 V for positive ionization and 4500 V for negative ionization mode, the declustering potential was set to 60 V, and the source temperature to 450 ^o^C for both modes. The curtain gas flow, nebulizer, and heater gas were set to 30, 40, and 45 units, respectively. The instrument performed one TOF MS survey scan (150 ms) and 15 MS/MS scans with a total duty cycle time of 2.4 s. The mass range in both modes was 50–1200 *m/z*. We controlled the acquisition of MS/MS spectra by data-dependent acquisition (DDA) function of the Analyst TF software (AB Sciex, Concord, Canada) with the following parameters: dynamic background subtraction, charge monitoring to exclude multiply charged ions and isotopes, and dynamic exclusion of former target ions for 9 s. Rolling collision energy spread was set whereby the software calculated the collision energy value to be applied as a function of *m/z*. Mass accuracy was maintained by the use of an automated calibrant delivery system interfaced to the second inlet of the DuoSpray source.

### Lipidomics data analysis

Raw data were converted to the mgf data format using proteoWizard software (2). The NIST MS PepSearch Program was used to search the converted files against LipidBlast libraries. The *m/z* width was determined by the mass accuracy of internal standards and were set to 0.001 for positive mode and to 0.005 for negative mode with an overall mass error of less than 2 parts per million. The minimum match factor used was set to 400. All raw data files were searched against the library of identified lipids based on mass and retention time using Multiquant 1.1.0.26 (ABsciex, Concord, Canada). Relative abundance of peak spectra was used for the analyses. Lipids that were identified in both positive and negative ion modes were initially analyzed separately for their relationship with outcome to ensure persistent results. As the relationship with outcome was not different in such lipids by ion modes, the values from positive mode were used in the final analysis. For lipid features with multiple adducts, the sum of spectral peaks from different adducts was used for the corresponding lipid. Identified lipids were quantified by normalizing against their respective internal standard. Quality control samples were used to monitor the overall quality of the lipid extraction and mass spectrometry analyses. The distributions of detected lipid species across the quality control samples indicated high reproducibility.

### RNA-sequencing and quantitative real-time RT-PCR (RT-qPCR)

RNA from liver of D70 (*n* = 3 per treatment group) and of D240 (*n* = 3 per treatment group) male rats was prepared using the Qiagen RNeasy kit (cat no. 74101), including optional on-column DNase digestion, according to the manufacturer’s instructions. RNA concentration was measured using Thermo Scientific’s Nanodrop and then sent to the Next Generation Sequencing Core at the University of Texas MD Anderson Cancer Center Science Park for RNA quality analysis, library prep and sequencing. RNA quality control was performed with the Agilent 2100 Bioanalyzer. The RNA Integrity Number (RIN) was determined for every sample and all samples used for sequencing libraries had RIN values > 8.4. Sequencing libraries were prepared using the Illumina Truseq stranded total RNA kit using 1000 ng of RNA per sample. Sequencing was performed using the HiSeq 3000 (Illumina).

To validate differentially expressed genes, frozen tissue from male rats was pulverized and processed with TRIzol (Invitrogen) per the manufacturer’s instructions. Isolated RNA was purified via ethanol precipitation and utilized for cDNA synthesis using the SuperScript III First-Strand Synthesis System (Life Technologies). 2 μg of RNA was incubated with RT reaction mix and RT enzyme mix for 10 min at 25 °C, followed by incubation at 50 °C for 30 min, at 85 °C for 5 min. After chilling on ice, 2U of E. Coli RNase H were added, followed by incubation at 37 °C for 20 minutes. Fast SYBR Green Master Mix (Applied Biosystems) and a Viia7 RT-PCR System (Life Technologies) were used for real-time RT-PCR measurement of mRNA levels via the 2^−ΔΔCt^ method (using *Gapdh* as the normalizer) under default reaction conditions. Primer sequences are provided in Supplementary Table 5.

### Transcriptomics data analysis

RNA-seq data yielded 29–37 million read pairs per sample. Data was mapped onto the rat genome UCSC build rn6 using hisat2, and gene expression was quantified using featureCounts against the GENCODE gene definitions. Gene expression was normalized using the Remove Unwanted Variation (RUVr)^71^ software. Differentially expressed genes were detected using DESeq2 with the Wald method, with significance achieved for FDR-adjusted *q*-value < 0.1 and fold change greater than or equal than 1.25× or lower than or equal to 0.8×. RNA-seq visualization using principal component analysis (PCA) and heatmaps were generated using the Python language scientific library. Enriched pathways were inferred using the over-representation analysis method (ORA), as implemented by the Molecular Signature Database (MSigDB)^72^ using the hypergeometric test and with significance achieved for FDR-adjusted *q*-value < 0.2. Enriched pathways were plotted as -−log10(*q*-value) using the GraphPad Prism software version 8.02. Targets of the EGR1 transcription factor were obtained using the Harmonizome Compendium; they specifically comprise ChIP-seq targets of EGR1 experimentally identified by ENCODE Consortium^73^, predicted based on sequence motifs by databases such as TRANSFAC or JASPAR^74,75^, or identified by hybrid methods such as Chip-X enrichment analysis^76^. We independently analyzed upregulated genes, downregulated genes, and all genes for the D240 EDC vs D240 vehicle signature. Overlaps were plotted using the GraphPad Prism 8.02 software.

### Chromatin immunoprecipitation (ChIP), ChIP-sequencing, and qPCR

Frozen liver tissue was pulverized, followed by cross-linking of proteins to DNA with 37% formaldehyde, and then incubation with 10X glycine to stop the reaction. Cross-linked tissue was homogenized, and centrifuged at 400 × *g* for 5 min at 4 °C. The cell pellet was resuspended with cell lysis buffer (PBS with 0.5 mM EDTA and 0.05% Triton X-100), incubated on ice for 15 min, followed by centrifugation at 2,500 × *g* for 5 min at 4 °C. The cell pellet was then resuspended with nuclear lysis buffer (1% SDS, 10 mM EDTA, 50 mM Tris-HCl at pH 8.1) and incubated on ice for 10 min. Chromatin was sonicated with a bioruptor (Diagenode, Denville, NJ) to obtain fragment sizes of 100–1000 bp or 100–300 bp for ChIP-qPCR and ChIP-seq, respectively.

ChIP was performed by incubating sheared chromatin with Magna ChIP^TM^ Protein A+G magnetic beads, and antibodies against H3K4me3 (Active Motif #39915; 1:100 dilution), H3K4me1 (Abcam ab8895; 1:100 dilution), H3K27ac (Abcam ab4729; 1:100 dilution) or H3K27me3 (Active Motif #39155; 1:100 dilution) overnight at 4 °C.The next day, the beads were washed with low salt wash buffer (0.1% SDS, 1% Triton X-100, 2 mM EDTA, 20 mM Tris-HCl pH 8, and 150 mM NaCl) high salt wash buffer (0.1% SDS, 1% Triton X-100, 2 mM EDTA, 20 mM Tris-HCl pH 8, and 500 mM NaCl), lithium chloride wash buffer (0.25 M LiCl, 1% IGEPAL CA630, 1% sodium deoxycholate, 1 mM EDTA, and 10 mM Tris-HCl pH 8), and TE buffer (20 mM Tris-HCl pH 8 and 1 mM EDTA) for 5 min each at 4 °C. Immunoprecipitated DNA was recovered from the beads by incubation with ChIP elution buffer (1% SDS, 1 mM EDTA, 50 mM NaHCO_3_, and 50 mM Tris-HCl pH 8) and proteinase K for 2 h at 62 °C with shaking, followed by incubation at 95 °C for 10 min. DNA was purified using the QIAquick PCR Purification kit (Qiagen) and DNA concentration was measured using a NanoDrop spectrophotometer (Thermo Scientific). ChIP-sequencing was performed by the Next Generation Sequencing Core at the University of Texas MD Anderson Cancer Center Science Park. Sequencing libraries were prepared using the Bioo Kit Option 2 protocol using 1.7–10 ng of DNA per sample. Sequencing was performed using the HiSeq 2500 (Illumina).

To validate genes with histone modification alterations, qPCR was performed using Fast Sybr Green Master Mix (Applied Biosystems) and a Viia7 RT-PCR System (Life Technologies) via the 2^−ΔΔCt^ method under default reaction conditions. Primer sequences are provided in Supplementary Table 5.

### Epigenomics data analysis

ChIP-seq in rat liver samples yielded 14–44 million single end reads per sample. Reads were trimmed for low quality basepairs using TrimGalore. Data was mapped to the rat genome build UCSC rn6 using the bowtie2 software. Duplicate reads were removed, then ChIP-seq track were prepared using bedtools, normalized to reads per million reads mapped (rpm). ChIP-seq tracks were visualized using the Integrative Genome Viewer software. Differential ChIP-seq regions were determined using the diffReps software using the G-test, with significance achieved for a fold change exceeding 1.5×, and an FDR-adjusted *q*-value < 0.01. Differential regions were annotated for nearby genes using BEDTOOLS; specifically, we considered genes with a differential region within 3 kb from its TSS. To annotate differential regions using enhancers, we first considered the Fantom5 enhancers compendium^27^, determined for a collection of mouse tissues, then derived corresponding regions on the rat genome using the UCSC liftOver tool^28^. Overall, we were able to identify 25527 enhancer regions on the rat genome with this approach. Overlap between rat enhancers and differential peaks was determined using BEDTOOLS. Venn diagram analysis of genes or enhancers associated with differential peaks was carried out using the Python language scientific library.

ChromHMM was used with the H3K4me1, H3K27ac, H3K4me3, and H3K27me3 ChIP-seq data to generate an epigenomic states partition of the rat liver epigenome. We down-sampled all ChIP-seq samples to 5 million deduplicated single-end reads to ensure ChromHMM calling will not be biased by different depth among samples. Epigenomic states were annotated based on the emission matrix following the approaches used by the Encode and the NIH Epigenome Roadmap consortia. Overlap of differential regions with epigenomic states was determined using BEDTOOLS. Odds-ratio enrichments for individual epigenomic states were then computed based on the cumulative genome-wise size of each epigenomic state. Odds-ratio were graphed using GraphPad Prism 8.02.

### Liver metabolomics

Frozen liver tissue (100 mg) from D70 (*n* = 5 per treatment group) and from D240 (*n* = 5 per treatment group) was submitted to the Metabolomics Core at BCM for targeted metabolomics analysis. Metabolites were extracted from crushed liver samples and a mouse liver pool was used for quality control. Twenty-five mg of crushed liver was used for the metabolic extraction. The extraction step started with the addition of 750 µL ice-cold methanol:water (4:1) containing 20 µL spiked internal standards to each tissue sample. Ice-cold chloroform and water were added in a 3:1 ratio for a final proportion of 1:4:3:1 water:methanol:chloroform:water. The organic (methanol and chloroform) and aqueous layers were mixed, dried and resuspended with 50:50 methanol: water. The extract was deproteinized using a 3 kDa molecular filter (Amicon ultracel-3K Membrane; Millipore Corporation, Billerica, MA) and the filtrate was dried under vacuum (Genevac EZ-2plus; Gardiner, Stone Ridge, NY). Prior to mass spectrometry, the dried extracts were resuspended in identical volumes of injection solvent composed of 1:1 water: methanol and were subjected to liquid chromatography-mass spectrometry.

For internal standards, high-performance liquid chromatography (HPLC)-grade acetonitrile, methanol, and water were procured from Burdick & Jackson (Morristown, NJ). Mass spectrometry-grade formic acid was purchased from Sigma-Aldrich (St Louis, MO). Calibration solution containing multiple calibrants in a solution of acetonitrile, trifluroacetic acid, and water was purchased from Agilent Technologies (Santa Clara, CA). Metabolites and internal standards, including N-acetyl Aspartic acid-d3, Tryptophan-15N2, Sarcosine-d3, Glutamic acid-d5, Thymine-d4, Gibberellic acid, Trans-Zeatine, Jasmonic acid, 15 N anthranilic acid, and testosterone-d3, were purchased from Sigma-Aldrich (St. Louis, MO).

Three LC- MS methods were used to separate metabolites. Method A: In ESI positive mode the HPLC column was waters X-bridge amide 3.5 µm, 4.6 × 100 mm (Waters, Milford, MA). Mobile phase A and B were 0.1% formic acid in water and acetonitrile, respectively. Gradient flow: 0–3 min 85% B; 3–12 min 30% B, 12–15 min 2% B, 16 min 95%B, followed by re-equilibration till the end of the gradient 23 min to the initial starting condition of 85% B. Flow rate of the solvents used for the analysis is 0.3 ml/min. Injection volume was 10 µL. Method B: In ESI negative mode the HPLC column was waters X-bridge amide 3.5 µm, 4.6 × 100 mm (Waters, Milford, MA). Mobile phase A and B were 20 mM ammonium acetate in water with pH 9.0 and 100% acetonitrile, respectively. Gradient flow: 0–3 min 85% B, 3–12 min 30% B, 12–15 min 2% B, 15–16 min 85% B followed by re-equilibration till the end of the gradient 23 min to the initial starting condition of 85% B. Flow rate of the solvents used for analysis is 0.3 ml/min. Injection volume was 10 µL. Method C: In ESI positive mode the HPLC column was Luna 3 µM NH2 100 A^0^ Chromatography column (Phenomenex, Torrance, CA). Mobile phase A and B were 20 mM ammonium acetate in water with pH 9.0 and 100% acetonitrile, respectively. Gradient flow: 0–3 min 85% B, 3–12 min 30% B, 12–15 min 2% B, 15–16 min 85% B followed by re-equilibration till the end of the gradient 23 min to the initial starting condition of 85% B. Flow rate of the solvents used for analysis is 0.3 ml/min. Injection volume was 10 µL.

For data acquisition through LC/MS analysis, 10 µL of suspended samples were injected and analyzed using a 6495 triple quadrupole mass spectrometer (Agilent Technologies, Santa Clara, CA) coupled to a HPLC system (Agilent Technologies, Santa Clara, CA) via Multiple reaction monitoring (MRM). Source parameters were as follows: gas temperature, 250 °C; gas flow, 14 l/min; nebulizer, 20 psi; sheath gas temperature, 350 °C; sheath gas flow, 12 l/min; capillary, 3000 V positive and 3000 V negative; nozzle voltage, 1500 V positive and 1500 V negative. Approximately 8–11 data points were acquired per detected metabolite. The data acquired using Agilent mass hunter software and data were analyzed using mass hunter quantitative analysis software.

### Quantification and statistical analysis

Student’s *t*-test (GraphPad Prism) was used to determine whether there were significant differences between VEH and BPA treatment groups for body weight, liver weight, serum ALT, serum lipids (measured by kits), liver gene expression (measured by real-time RT-qPCR), and liver histone modifications (measured by ChIP-qPCR). *P* ≤ 0.05 was considered significant.

For the lipidomics analysis, after data acquisition, the missing values for lipids were imputed using the K nearest-neighbor method. Then the data were log2 transformed followed by normalization using the day median normalization. The compound-by-compound two-sided parametric *t*-test was applied, followed by false discovery rate (FDR) correction. Significance was assigned to fdr-adjusted *q*-values < 0.25.

For the metabolomics analysis, the data were log2-transformed and normalized with internal standards on a per-sample, per-method basis. For every individual metabolite we computed the mean value and the standard deviation within vehicle-treated animals. Next, we determined a z-score for each metabolite in each EDC-exposed rat, reflecting its standard deviation from the median values observed in vehicle-treated animals. Employing the approaches pioneered by the CBioPortal^35^ resource and its widely-used Oncoprint data exploration and visualization tool, we identified and selected metabolites increased by at least 1.5 standard deviations in at least 3 EDC-exposed animals and down in none, or decreased by at least 1.5 standard deviations in at least 3 EDC-exposed animals and up in none.

We next integrated hybrid metabolites and gene signatures using the hybrid metabolite/transcriptomics pathway compendium WikiPathways^36^. We extended the metabolites-only MSEA enrichment method to hybrid metabolites/genes signatures using hypergeometric distribution with significance assigned to fdr-adjusted *q*-values < 0.25. We implemented this approach in our MetSEA (metabolomics set enrichment analysis) software.

### Reporting summary

Further information on research design is available in the Nature Research Reporting Summary linked to this article.

## Supplementary information

Supplementary Information

Reporting Summary

## Data Availability

The datasets generated during the current study have been deposited in public repositories. ChIP-seq data have been deposited in the NCBI Gene Expression Omnibus (GEO) with the accession code GSE130409. RNA-seq data have been deposited in the NCBI GEO with the accession code GSE130434. The metabolomics and lipidomics data are available at the NIH Common Fund’s National Metabolomics Data Repository (NMDR) website (https://www.metabolomicsworkbench.org), the Metabolomics Workbench, where it has been assigned Project ID PR000890 (10.21228/M8ND7K). This repository is supported by the NIH grant U2C-DK119886. The source data underlying Figs. 1b, c, 3a, b, e–g, and 4c, and Supplementary Figs. 1a–c, e–f, 3c, 4a–f, and 5a, b are provided as a Source Data file.

## Source Data

Source Data
