# Supplementary material for: Epigenome environment interactions accelerate epigenomic aging and unlock metabolically restricted epigenetic reprogramming in adulthood
Source: Nat Commun. 2020 May 8;11:2316. doi: 10.1038/s41467-020-15847-z (PMC7210260; doi:10.1038/s41467-020-15847-z)
Supplement: Supplementary file 1 — Supplementary Information [file 41467_2020_15847_MOESM1_ESM.pdf]

Supplementary Information

Epigenome Environment Interactions Accelerate Epigenomic Aging and Unlock Metabolically Restricted Epigenetic Reprogramming in Adulthood

Treviño et al.

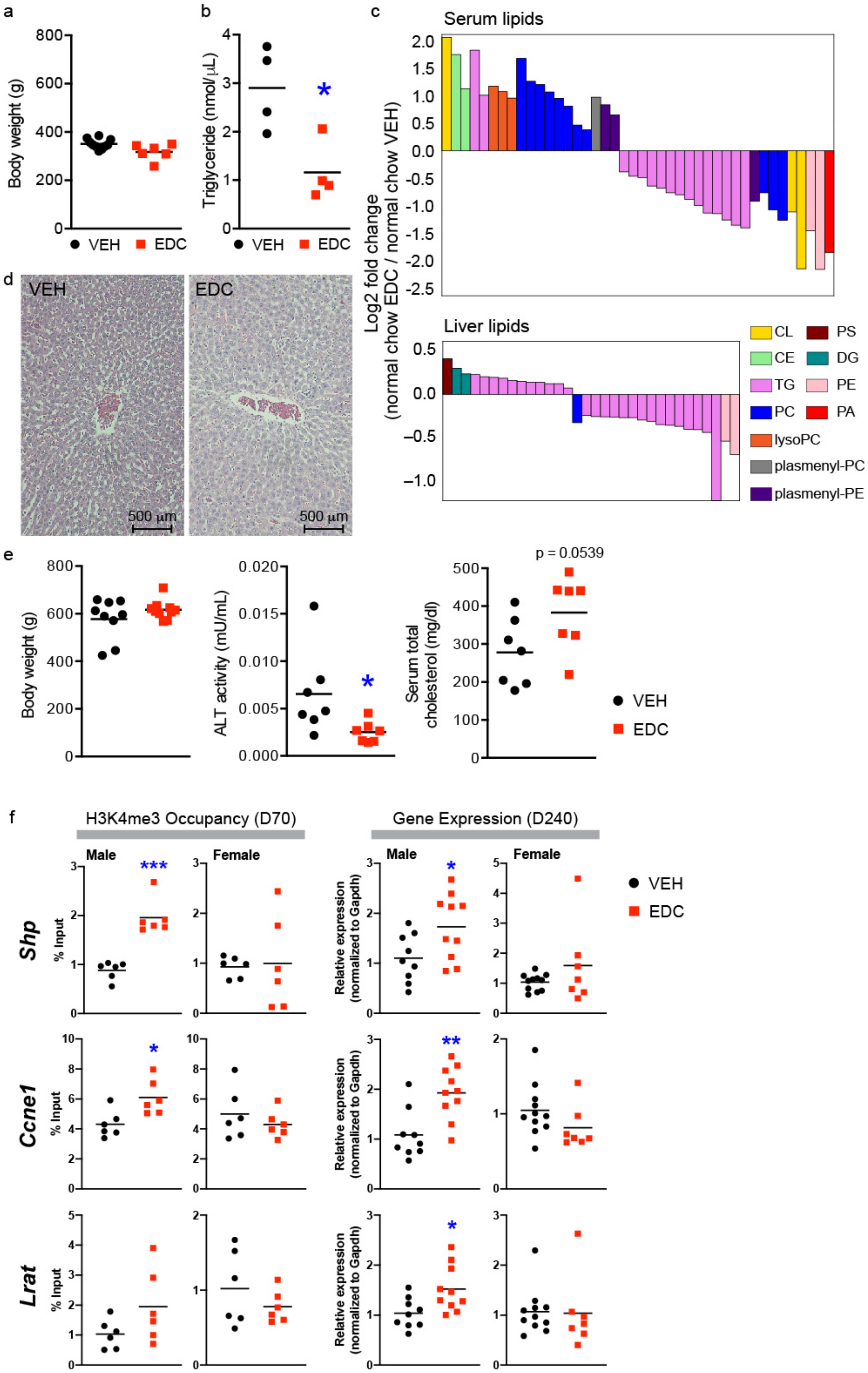

Supplementary Figure 1. Impact of EDC exposure on body weight, metabolic parameters, liver phenotype, and sexually dimorphic reprogramming

a. Body weight of D70 animals treated with VEH (black circles) or EDC (red squares) during PND1-5. N= 7 biologically independent animals for VEH and N = 6 biologically independent animals for EDC.

b. Serum triglycerides of D70 animals treated with VEH (black circles) or EDC (red squares) during early life. N= 4 biologically independent animals per treatment. The *p* value generated by t *test* is indicated. * $p < 0.05$.
c. Targeted serum and liver lipidomic analysis of EDC- vs VEH-exposed animals at D70. N = 5 biologically independent animals per treatment. Abbreviations are phospholipids [phosphatidylcholine (PC), phosphatidylserine (PS), phosphatidylethanolamine (PE), phosphatidic acid (PA)], cardiolipin (CL), cholesteryl ester (CE), diacylglycerol (DG), and triglyceride (TG). Lipid levels are reported as significantly ($q < 0.25$) increased in EDC-exposed animals on Western-style diet compared to VEH-exposed animals fed the same diet. Each bar is a different specific lipid within a color-coded class for each lipid.
d. Representative H&E stained sections of a vehicle-exposed animal (left) and an EDC-exposed animal (right) at D70. Scale bar = 500 μm.
e. Body weight (VEH, N =9 biologically independent animals; EDC, N = 10 biologically independent animals), serum alanine aminotransferase (ALT) activity (N = 7 biologically independent animals per treatment; * $p < 0.05$), and total serum cholesterol (N = 7 biologically independent animals per treatment; p =0.0539). Black circles represent VEH and red squares represent EDC animals. The *p* value generated by t *test* is indicated.
f. H3K4me3 occupancy at EDC-reprogrammed genes (*Shp*, *Ccne1*, and *Lrat*) at D70 as measured by ChIP-qPCR (left). N = 6 biologically independent animals per treatment. *Shp*, *Ccne1*, and *Lrat* gene expression at D240 as measured by RT-qPCR (right). N = 11 biologically independent animals for VEH and N = 10 biologically independent animals for EDC. Black circles represent VEH and red squares represent EDC animals. The *p* values generated by t *test* are indicated. * $p < 0.05$; ** $p < 0.01$; *** $p < 0.001$

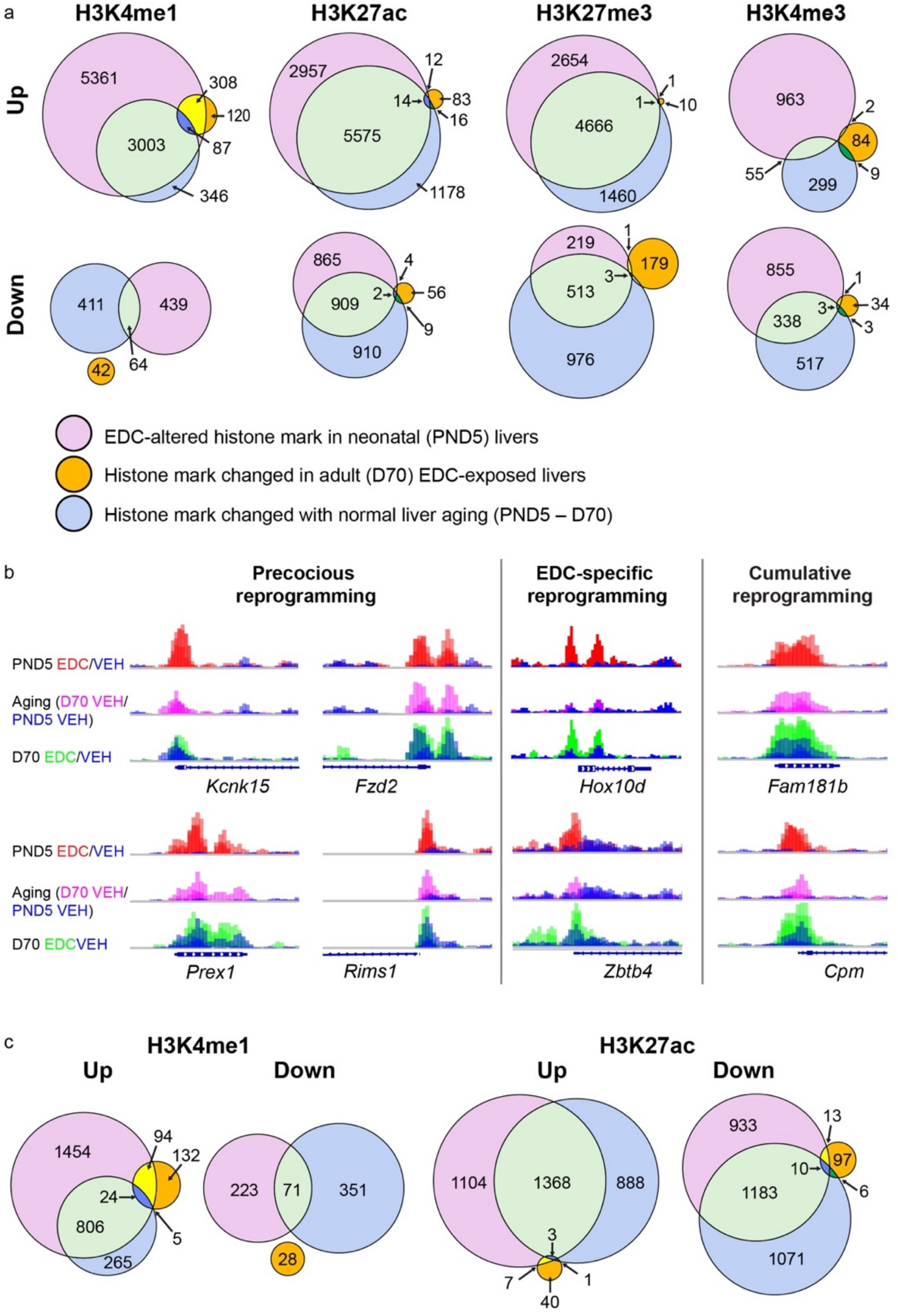

Supplementary Figure 2. Robust reprogramming of H3K4me1, H3K27ac, and H3K27me3 in response to EDC exposure

a. Venn diagrams for H3K4me1, H3K27ac, H3K27me3, and H3K4me3 focused on histone modifications associated with target genes, defined as having differential peak calls within +/- 3 kb of the transcriptional start site (TSS), using the GENCODE gene model and the BEDTOOLS software (N = 3 biologically independent animals per timepoint/treatment). Both increased, and decreased, peak calls are shown as Up and Down, respectively.

b. The integrated genome viewer (IGV) provides representative examples of each type of reprogramming for increased H3K4me1 at target genes. Precocious Reprogramming is illustrated by *Kcnk15, Fzd2, Prex1,* and *Rims1*, EDC-specific Reprogramming illustrated by *Hox10d* and *Zbtb4, and* Cumulative Reprogramming illustrated by *Fam181b* and *Cpm*. The data shown on the y-axis are the ChIP-seq read counts normalized to 1 million mapped reads.
c. Venn diagrams for H3K4me1 and H3K27ac focused on modifications at enhancers. Data from the comprehensive mouse enhancer maps of multiple cell types from the Fantom5 consortium were used with the UCSC liftOver tool to map mouse enhancers onto the rat genome: this approach identified 25527 rat enhancers. The majority of changes observed for enhancers were a result of Precocious Reprogramming.

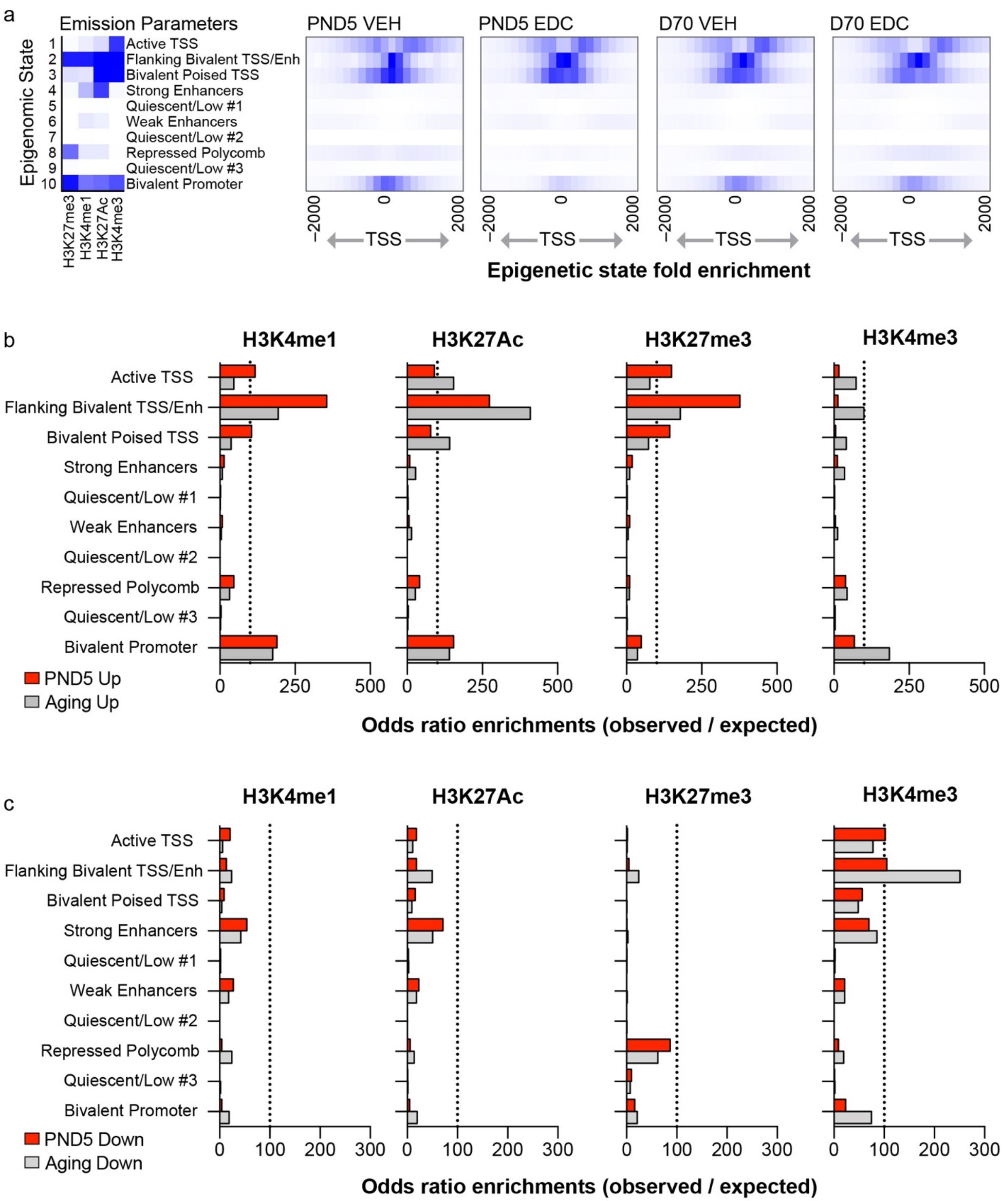

Supplementary Figure 3. EDC-mediated reprogramming of specific chromatin states

a. Epigenomic state enrichment seen around the transcription start site (TSS) in response to EDC exposure at PND5 and at D70. Epigenome states were inferred by integrating data for all histone modifications generated at both PND5 and D70 time points, in both EDC- and vehicle (VEH)-exposed livers, and further annotated based on specific combinations of marks following the Encode approach (Emission Parameters, left). Epigenome state enrichment for each individual state is shown around the TSS (right).

b. ChromHMM odds ratio enrichment analysis for increases in H3K4me1, H3K27ac, H3K27me3, and H3K4me3 associated with EDC exposure (PND5 BPA vs PND5 VEH) and with normal aging (D70 VEH versus PND5 VEH). For each mark and epigenomic state, we first determined the overlap and then the odds-ratio enrichment $\geq$ 100 (dotted line).
c. ChromHMM odds ratio enrichment analysis for decreases in H3K4me1, H3K27ac, H3K27me3, and H3K4me3 associated with EDC exposure (PND5 BPA vs PND5 VEH) and with normal aging (D70 VEH versus PND5 VEH). For each mark and epigenomic state, we first determined the overlap and then the odds-ratio enrichment $\geq$ 100 (dotted line).

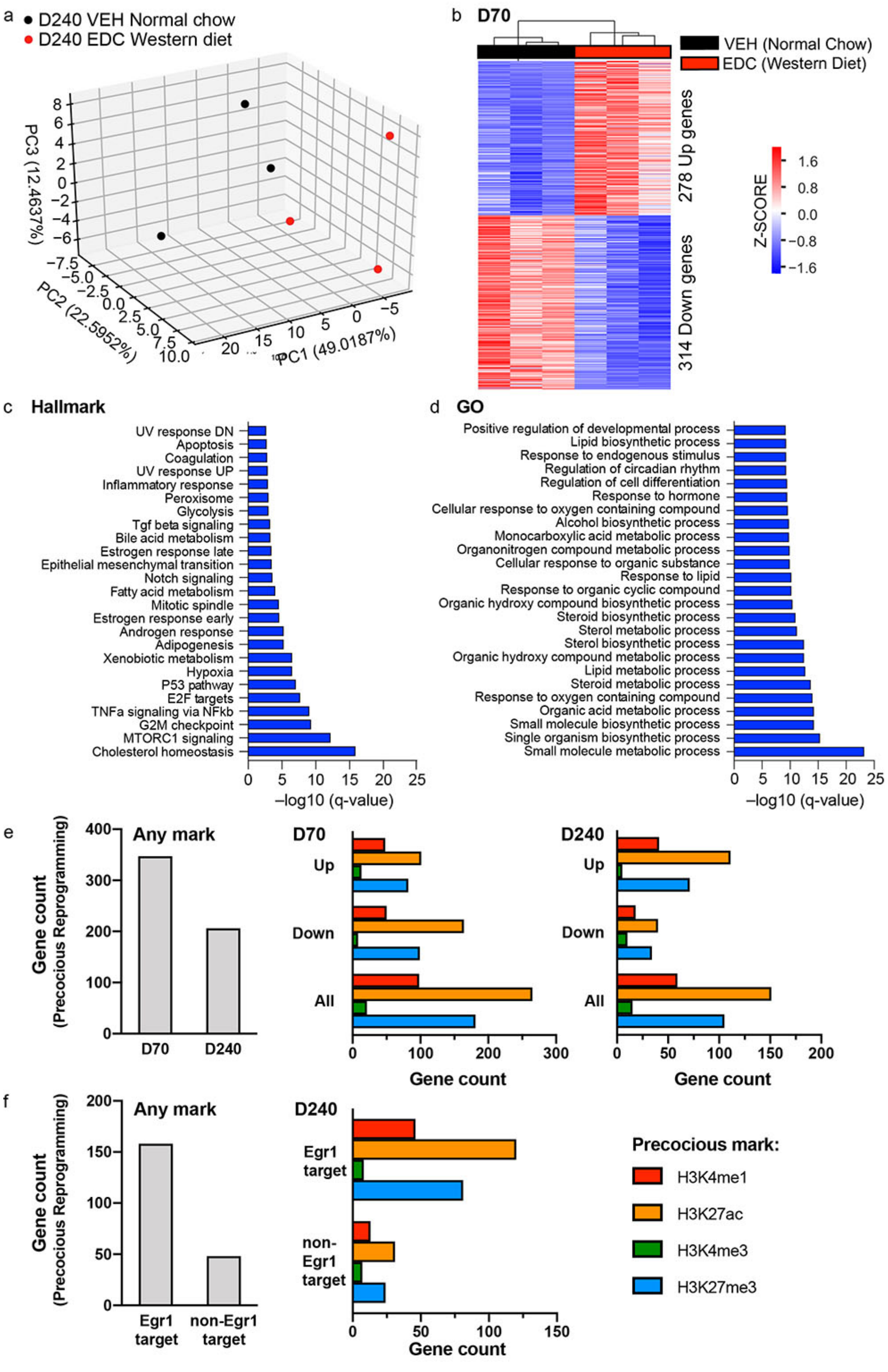

Supplementary Figure 4. Transcriptomic and epigenomic analyses reveals reprogrammed targets at D70 and D240

a. Principal component analysis (PCA) using expressed genes from vehicle-exposed (black dots) or from EDC-exposed (red dots) livers from animals fed a Western-style diet. N = 3 biologically independent animals per treatment.

b. Heat map showing RNA-seq data from vehicle- (black bar) and EDC- (red bar) exposed livers from D70 animals. Differentially regulated genes were identified using RUVr normalization and DESeq differential gene analysis, with significance achieved at q-value < 0.1 and fold change exceeding 1.25x. N = 3 biologically independent animals per treatment.
c-d. Overrepresentation analysis (ORA) revealed significant enrichment for genes in metabolism-associated pathways in the reprogrammed livers of animals at D70. ORA of differentially expressed genes was performed using a hypergeometric test, with significance achieved at q < 0.2, against the Hallmark gene set compendium (c) and the GO database (d).
e. Identification of differentially expressed genes at D70 (347/592) and D240 (206/431) that were targets for Precocious Reprogramming for any mark cumulatively (left) and for each individual mark (right).
f. Identification of differentially expressed genes at D240 that were targets of Precocious Reprogramming for any mark (left) and for each individual mark (right), and that were also targets for EGR1 (158/206).

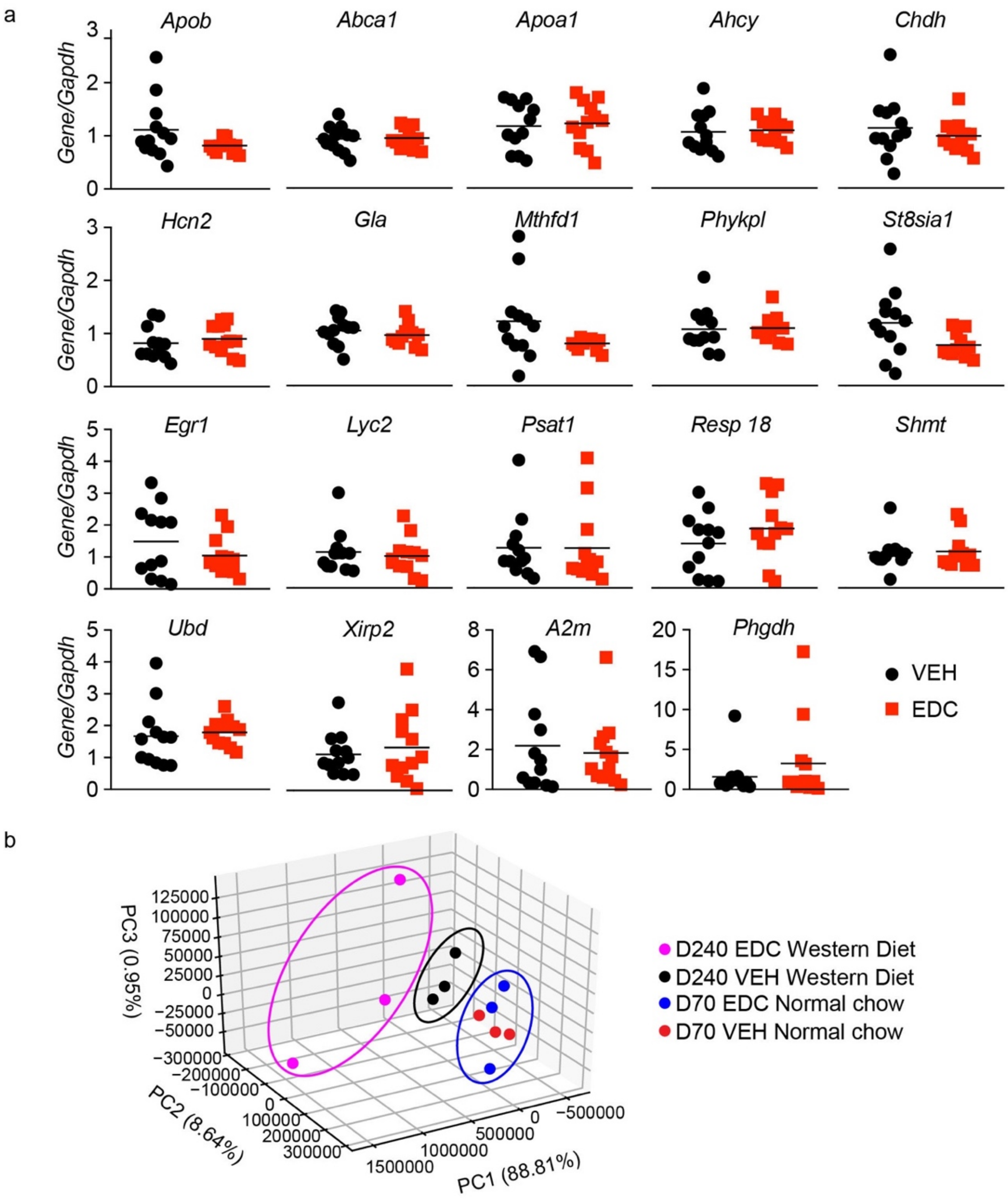

Supplementary Figure 5. Effects of EDC-mediated reprogramming are revealed in the setting of a Western-style diet

a. Quantitative RT-PCR analysis showing no change in expression of reprogrammed genes in the livers of one-year old EDC-exposed animals maintained on a normal chow diet (EDC; red squares) compared to vehicle-exposed animals (VEH; black dots) shown by RT-qPCR. N = 12 biologically independent animals per treatment.

b. Principal component analysis (PCA) using expressed genes from vehicle-exposed (red dots) or EDC-reprogrammed (blue dots) liver from adult animals maintained on normal chow (D70), or from vehicle-exposed (black dots) or EDC-reprogrammed (magenta dots) liver from animals both fed a Western-style diet (D240) using the same principal components as in Supplementary Fig. 4a. N = 3 biologically independent animals per timepoint/treatment.

Supplementary Table 1: Top 20 Up and Down Genes D240

Gene (UP)	Log2 Fold Change	-log10 (q-value)	Gene (DOWN)	Log2 Fold Change	-log10 (q-value)
LOC103690054	4.92	12.25	Lilrb4	-1.29	2.66
Xirp2	2.78	1.87	Slc13a5	-1.29	5.49
A2m	2.63	7.12	AABR07048474.1	-1.31	12.25
LOC685203	2.61	2.60	Chi3l1	-1.34	1.40
Tmem200a	2.39	1.58	Gpat3	-1.45	1.50
Gla	2.24	5.00	Cxcl9	-1.46	1.27
Psat1	2.14	1.52	Sctr	-1.57	1.46
Ciart	2.03	1.70	Nfs1	-1.59	2.49
Lyc2	1.94	22.81	AABR07047899.1	-1.64	9.97
Phgdh	1.92	1.43	AC123346.1	-1.69	1.52
RGD1560242	1.82	1.81	LOC100360095	-1.72	15.99
Dbp	1.76	1.80	Mboat1	-1.80	1.24
AABR07054614.1	1.69	1.36	RGD1305184	-1.92	4.82
Kcnj15	1.69	4.33	RGD1566134	-2.15	9.36
LOC108348081	1.66	1.34	St8sia1	-2.20	1.61
Hist1h4b	1.60	1.08	LOC100912599	-2.53	4.40
Thrsp	1.59	2.48	Phykpl	-2.62	1.57
Egr1	1.58	4.89	Resp18	-3.75	1.95
AC105662.1	1.55	1.39	Hcn2	-3.78	3.29
Hsd17b2	1.52	1.22	Ubd	-7.37	3.72

Supplementary Table 2: Top 25 Genes by Absolute Loading Value (D240 BPA vs VEH)

Gene	PC1	Gene	PC2	Gene	PC3
Ubd	1.83	Igfbp2	0.99	LOC100909524	0.66
Cxcl9	1.06	Bcl6	-0.69	LOC29811	0.60
Gpnmb	0.91	Stac3	-0.65	LOC100911581	0.58
RT1-Db1	0.91	Slc34a2	-0.65	Ubd	0.56
Cd74	0.88	Cish	0.63	Echs1	-0.55
RT1-Da	0.88	LOC100909524	0.59	LOC100911186	0.50
Gbp2	0.88	Lox	-0.56	Grcc10	0.47
Dbp	-0.85	Angptl4	-0.56	Timd2	-0.45
RGD1305184	0.83	LOC100912380	-0.55	Socs2	0.44
Hsd17b2	-0.81	G0s2	-0.54	Selenbp1	0.44
Timd2	-0.79	LOC100910308	0.53	Cish	0.42
Hk3	0.78	LOC100360095	-0.52	Bcl6	-0.42
Gbp6	0.77	Scd	-0.51	Fras1	-0.40
AABR07047899.1	0.76	G6pc	-0.49	Hdc	-0.40
Lyz2	0.75	Sds	0.49	Dusp1	0.38
Marco	0.74	Upp2	0.49	Aldh1a7	0.37
Cdh17	0.74	Plin2	-0.49	Slc27a5	0.36
Phgdh	-0.73	Ubd	-0.49	Map1b	-0.35
Gdp6	0.71	Aldh1a7	-0.49	Cyp4a2	0.30
LOC29811	0.70	Ces2e	0.46	LOC108348062	-0.29
Slc11a1	0.69	LOC103690054	0.45	LOC100912481	-0.29
Ncf1	0.67	Mybl1	-0.44	Cdh17	-0.29
Lilrb3	0.66	Selenbp1	0.44	LOC103689931	0.27
Psmb9	0.65	Cyp2c12	-0.44	Dpt	0.27
Lyc2	-0.65	Nab2	0.43	Hhex	0.26

Supplementary Table 3: Top 20 Up and Down Genes D70

Gene (UP)	Log2 Fold Change	-log10 (q-value)	Gene (DOWN)	Log2 Fold Change	-log10 (q-value)
Pdcd5	5.03	1.41	Per1	-2.02	18.96
Tmem200a	4.31	2.56	Zfp354a	-2.06	1.03
Ndufa1	4.23	1.69	Cyp2c24	-2.13	2.61
Igfn1	3.39	1.36	Lpin1	-2.13	32.95
AABR07001512.1	2.90	4.19	Exoc7	-2.19	1.37
Tbcb	2.63	1.23	Vxn	-2.20	6.51
Arntl	2.56	85.47	LOC103690018	-2.35	2.94
AC105662.1	2.49	10.35	LOC100361492	-2.36	7.06
Ube2c	2.36	1.38	Noct	-2.40	1.00
Lonrf3	2.36	7.46	Upp2	-2.40	26.17
AABR07028989.1	2.34	1.78	Usp2	-2.87	20.62
Mme	2.32	5.02	Hsd17b2	-2.90	206.11
Ccnb1	2.31	8.00	Sdr16c6	-2.97	4.23
Sfxn2	2.30	1.93	Ciart	-3.40	2.66
Mmd2	2.19	6.01	LOC100911660	-3.46	8.02
Slc6a6	2.19	46.35	Per3	-3.59	79.01
RGD1560010	2.16	4.63	LOC103690054	-4.80	9.39
Rrm2	2.08	2.56	LOC103690108	-5.11	1.86
Map3k7	2.06	1.07	Dbp	-5.29	176.24
LOC100910308	1.97	6.19	Ubd	-7.03	24.12

Supplementary Table 4: MetSEA Analysis of Liver Reprogramming in Response to Western-style Diet

Pathway	q-value	Number of Metabolites	Overlapping Metabolites	Number of Genes	Overlapping Genes
Selenium Micronutrient Network	0.005	3	L-METHIONINE, L-TRYPTOPHAN, HYPOXANTHINE	7	APOB, ALB, INSR, ABCA1, IL1B, SELP, APOA1
Folate Metabolism	0.005	2	GLYCINE, L-METHIONINE	7	APOB, ALB, INSR, ABCA1, IL1B, AHCY, APOA1
Vitamin B12 Metabolism	0.022	1	L-METHIONINE	6	APOB, ALB, INSR, ABCA1, IL1B, APOA1
Exercise-induced Circadian Regulation	0.022	0		8	HERPUD1, HSPA8, CRY2, PER2, PER1, NR1D2, CBX3, KLF9
Statin Pathway	0.050	0		6	SOAT1, APOB, ABCA1, FDFT1, APOA1, APOA2
Trans-sulfuration and one carbon metabolism	0.055	2	GLYCINE, L-METHIONINE	5	GCLC, PSAT1, PHGDH, AHCY, SHMT1
Serotonin Transporter Activity	0.080	1	L-TRYPTOPHAN	2	IL1B, IL1R1
One Carbon Metabolism	0.119	3	GLYCINE, L-METHIONINE, BETAINE	3	CHDH, AHCY, SHMT1
One carbon donor	0.119	4		0	
Amino Acid metabolism	0.131	6		6	IARS, ADH4, ASNS, PCK1, GLUL, OAT

Supplementary Table 5: Primer Sequences for RT-qPCR and ChIP-qPCR

Real-time RT-qPCR

Gene name	Forward primer	Reverse primer
EGR1	GAGTGTGCCCTCAGTAGCTT	CAATTGCATCTCGGCCTTGG
GAPDH	ATGACTCTACCCACGGCAAG	CTGGAAGATGGTGATGGGTT
SHMT1	AGTTCTCCGAGGCAATCAGC	TCAATGCTGGGGTTGAGACC
CHDH	ATCATGCCCAGTGTGGTCAG	GTCTTAACGCTGGGTGTCCA
MTHFD1	TGGTGTTCTTCGTGGGTAGC	CGATTCCTGATTTGCGCGG
AHCY	GCCTGTAAGGAGGGCAACAT	TCTGTTCAAAGTGCCGACCA
APOB	GGAGCAGTATTCCGCCAGTG	TGGACTCCTTCTGCTTGCAC
ABCA1	CCTGAAGCCAGTCATGACAAAAC	CCCCCTAAGCTGTCAAGCAAC
APOA1	TGGTCTTCCTGACAGGTTGC	GTGGCGAAATCCTTCACCCT
SHP	GCAGCACTGCCTGGAGTC	GTGTGCAATGTGGCAGGA
CCNE1	ATTCGCCATGGTTATCCGGG	CTTGGGCTTTGTCCAGCAAG
LRAT	AGCGGACATCCTCGTCAATC	TCGTGAAACTTCTCAGCCTGC
HCN2	ATCCTCAGTCTGCTGCGGTTG	TGTGGAAAATCTCCTCCCATTGGT
GLA	CCCGAGAGGGATTCAAAGGG	AGCCTTTGCTGTGGACGTAA
PHYKP1	CCACTACCCAAGCTGTGTCA	ATTGCCACCAAACGTGTTGAA
ST8SIA1	TGTCATGCGGTGTAACCTTC	GGGTTAGCTGTCACCAACTGA
LYC2	AAGGCATTCGAGCATGGGTG	TAGAAGTGCACTGCGGTCAG
PSAT1	AGCTCAGCTCCGTCAAATCC	GCTCCACCGGACACACATAA
RESP18	CAAGGACTCGTTGCCTCAGT	CCAGAACATGCCTTGGGGTA
UBD	GCTTCCTGCGTCTGTGTTGT	TGTCACTCATGGTGGTGTCAA
XIRP2	GACTTCAACCTGCAATGCCT	GGATTGCCTCCTGCTCAGAT
A2M	GCTCTCCGCATCCCTGATTT	TCTTCTTCCCGTTGATCCGC
PHGDH	GCCCACTATGATCGGCCTAC	CGTCAGACACCTTGGAGGTC

ChIP-qPCR

Gene name	Forward primer	Reverse primer
SHP	GCATTGTGTGTGTGTGTATGG	CCCTCTCTACGGAGTGCTATT
CCNE1	TCCCCTGAGGTATCAGCTCG	CTGCCTCAGTGTCCCTTTCT
LRAT	CTGCAGCCCCTAAACCACTT	CCCAAAATCAGGCAGGGGAT
