# Supplementary material for: Epigenome environment interactions accelerate epigenomic aging and unlock metabolically restricted epigenetic reprogramming in adulthood
Source: Nat Commun. 2020 May 8;11:2316. doi: 10.1038/s41467-020-15847-z (PMC7210260; doi:10.1038/s41467-020-15847-z)
Supplement: Supplementary file 2 — Reporting Summary [file 41467_2020_15847_MOESM2_ESM.pdf]

nature research

Corresponding author(s): Cheryl Lyn Walker

Last updated by author(s): Dec 19, 2019

Reporting Summary

Nature Research wishes to improve the reproducibility of the work that we publish. This form provides structure for consistency and transparency in reporting. For further information on Nature Research policies, see Authors & Referees and the Editorial Policy Checklist .

Statistics

For all statistical analyses, confirm that the following items are present in the figure legend, table legend, main text, or Methods section.

n/a	Confirmed	
☐	☒	The exact sample size (n) for each experimental group/condition, given as a discrete number and unit of measurement
☐	☒	A statement on whether measurements were taken from distinct samples or whether the same sample was measured repeatedly
☐	☐	The statistical test(s) used AND whether they are one- or two-sided<br>*Only common tests should be described solely by name; describe more complex techniques in the Methods section.*
☐	☒	A description of all covariates tested
☐	☒	A description of any assumptions or corrections, such as tests of normality and adjustment for multiple comparisons
☐	☒	A full description of the statistical parameters including central tendency (e.g. means) or other basic estimates (e.g. regression coefficient) AND variation (e.g. standard deviation) or associated estimates of uncertainty (e.g. confidence intervals)
☒	☐	For null hypothesis testing, the test statistic (e.g. F, t, r) with confidence intervals, effect sizes, degrees of freedom and P value noted<br>*Give P values as exact values whenever suitable.*
☒	☐	For Bayesian analysis, information on the choice of priors and Markov chain Monte Carlo settings
☒	☐	For hierarchical and complex designs, identification of the appropriate level for tests and full reporting of outcomes
☒	☐	Estimates of effect sizes (e.g. Cohen's d, Pearson's r), indicating how they were calculated

Our web collection on statistics for biologists contains articles on many of the points above.

Software and code

Policy information about availability of computer code

Data collection	ImageQuant TL was used to quantify band density of western blots.
Data analysis	Sequencing data quality was assessed using FastQC 0.11.8 and low quality basepairs were removed using TrimGalore 0.5.0. RNA-Seq samples were aligned to the genome build UCSC Rn6 using HISAT 2.1.0. Differential RNA-Seq genes were inferred using RUVSeq 1.16.1 and DESeq2 1.22.2 as implemented in the R statistical system 3.5.2. Differential ChIP-Seq analysis was performed using diffReps 1.55.6. Heatmaps, PCA and volcano plots were generated using the Python packages matplotlib 2.2.3 and seaborn 0.9.0. Transcriptomic based pathway analysis was carried out using the Python scientific library scipy 1.1.0. Metabolomics pathway analysis were generated using WikiPathways and the Python scientific library scipy 1.1.0. Signal tracks were generated using bedtools 2.27.1 and displayed using Integrated Genome Viewer 2.4.17. Circos plots were generated using circos 0.69-6. ChIP-Seq venn diagrams were created using the Python library matplotlib-venn 0.11.5. Chromatin epigenomic maps were generated using ChromHMM 1.18.

For manuscripts utilizing custom algorithms or software that are central to the research but not yet described in published literature, software must be made available to editors/reviewers. We strongly encourage code deposition in a community repository (e.g. GitHub). See the Nature Research guidelines for submitting code & software for further information.

Data

Policy information about availability of data

All manuscripts must include a data availability statement. This statement should provide the following information, where applicable:

- Accession codes, unique identifiers, or web links for publicly available datasets
- A list of figures that have associated raw data
- A description of any restrictions on data availability

The datasets generated during the current study have been deposited in public repositories. ChIP-seq data have been deposited in the NCBI Gene Expression Omnibus (GEO) with the accession code GSE130409 (https://www.ncbi.nlm.nih.gov/geo/query/acc.cgi?acc=GSE130409). RNA-seq data have been deposited in the NCBI GEO with the accession code GSE130434 (https://www.ncbi.nlm.nih.gov/geo/query/acc.cgi?acc=GSE130434). The metabolomics and lipidomics data are available at the NIH Common Fund's National Metabolomics Data Repository (NMDR) website (https://www.metabolomicsworkbench.org), the Metabolomics

Workbench, where it has been assigned Project ID PR000890. The data can be accessed directly via the Project DOI doi:10.21228/M8ND7K. This repository is supported by the NIH grant U2C-DK119886. The source data underlying Figs 1b-c, 3a-b, e-g, and 4c, and Supplementary Figs 1a-c, e-f, 3c, 4a-f, and 5a-b are provided as a Source Data file.

Field-specific reporting

Please select the one below that is the best fit for your research. If you are not sure, read the appropriate sections before making your selection.

- [x] Life sciences
- [ ] Behavioural & social sciences
- [ ] Ecological, evolutionary & environmental sciences

For a reference copy of the document with all sections, see nature.com/documents/nr-reporting-summary-flat.pdf

Life sciences study design

All studies must disclose on these points even when the disclosure is negative.

Sample size	No statistical method was used to predetermine sample size.
Data exclusions	No data were excluded.
Replication	RNA-sequencing data from 3 animals per treatment were validated with a larger cohort of animals using qRT-PCR. ChIP-sequencing data from 3 animals per treatment group were validated with a larger cohort of animals via ChIP-qPCR. Immunoblot analyses were performed to validate the observed increase in EGR1 gene expression.
Randomization	Neonatal rats were selected randomly for experimental treatment within each litter.
Blinding	The investigators were not blinded to the sample ID during experiments and data analysis because the data were collected objectively via sequencing (for ChIP- and RNA-seq) and mass spectrometry (for lipidomics and metabolomics).

Reporting for specific materials, systems and methods

We require information from authors about some types of materials, experimental systems and methods used in many studies. Here, indicate whether each material, system or method listed is relevant to your study. If you are not sure if a list item applies to your research, read the appropriate section before selecting a response.

Materials & experimental systems

n/a	Involved in the study	
☐	☒	Antibodies
☒	☐	Eukaryotic cell lines
☒	☐	Palaeontology
☐	☒	Animals and other organisms
☒	☐	Human research participants
☒	☐	Clinical data

Methods

n/a	Involved in the study	
☐	☒	ChIP-seq
☒	☐	Flow cytometry
☒	☐	MRI-based neuroimaging

Antibodies

Antibodies used	EGR1 (44D5) rabbit monoclonal antibody from Cell Signaling catalog number 4154S (immunoblot)
Validation	Antibody was validated as described on the vendor's website.

Animals and other organisms

Policy information about studies involving animals; ARRIVE guidelines recommended for reporting animal research

Laboratory animals	Male and female Sprague Dawley rats were treated with vehicle or EDC post-natally (within days 1-5). Samples were harvested on postnatal day 5, day 70, day 240, or day 360 (1 yr). Reprogramming was not observed in females (see Supplementary Figure 1). Therefore, most of the data reported in the manuscript is derived from males.
Wild animals	This study did not involve wild animals.
Field-collected samples	This study did not involve field-collected samples.
Ethics oversight	Experimental procedures were approved by the Institutional Care and Use Committee at Texas A&M Institute of Biosciences & Technology.

Note that full information on the approval of the study protocol must also be provided in the manuscript.

ChIP-seq

Data deposition

☒ Confirm that both raw and final processed data have been deposited in a public database such as GEO.

☒ Confirm that you have deposited or provided access to graph files (e.g. BED files) for the called peaks.

Data access links<br>*May remain private before publication.*	ChIP-seq data have been deposited in the NCBI Gene Expression Omnibus (GEO) with the accession code GSE130409 (https://www.ncbi.nlm.nih.gov/geo/query/acc.cgi?acc=GSE130409).
Files in database submission	Sample_10_VEH_2_K4me3.fastq.gz<br>Sample_11_VEH_3_K4me3.fastq.gz<br>Sample_12_VEH_4_K4me3.fastq.gz<br>Sample_16_BPA_2_K4me3.fastq.gz<br>Sample_17_BPA_3_K4me3.fastq.gz<br>Sample_18_BPA_4_K4me3.fastq.gz<br>Sample_19_VEH_2_K4me1.fastq.gz<br>Sample_20_VEH_3_K4me1.fastq.gz<br>Sample_21_VEH_4_K4me1.fastq.gz<br>Sample_25_BPA_2_K4me1.fastq.gz<br>Sample_26_BPA_3_K4me1.fastq.gz<br>Sample_27_BPA_4_K4me1.fastq.gz<br>Sample_28_VEH_2_K27ac.fastq.gz<br>Sample_29_VEH_3_K27ac.fastq.gz<br>Sample_30_VEH_4_K27ac.fastq.gz<br>Sample_34_BPA_2_K27ac.fastq.gz<br>Sample_35_BPA_3_K27ac.fastq.gz<br>Sample_36_BPA_4_K27ac.fastq.gz<br>Sample_37_VEH_2_K27me3.fastq.gz<br>Sample_38_VEH_3_K27me3.fastq.gz<br>Sample_39_VEH_4_K27me3.fastq.gz<br>Sample_43_BPA_2_K27me3.fastq.gz<br>Sample_44_BPA_3_K27me3.fastq.gz<br>Sample_45_BPA_4_K27me3.fastq.gz<br>Sample_BPA_14_H3K27ac.fastq.gz<br>Sample_BPA_14_H3K4me1.fastq.gz<br>Sample_BPA_14_K27me3.fastq.gz<br>Sample_BPA_14_K4me3.fastq.gz<br>Sample_BPA_23_H3K27ac.fastq.gz<br>Sample_BPA_23_H3K4me1.fastq.gz<br>Sample_BPA_23_K27me3.fastq.gz<br>Sample_BPA_23_K4me3.fastq.gz<br>Sample_BPA_25_H3K27ac.fastq.gz<br>Sample_BPA_25_H3K4me1.fastq.gz<br>Sample_BPA_25_K27me3.fastq.gz<br>Sample_BPA_25_K4me3.fastq.gz<br>Sample_VEH_27_H3K27ac.fastq.gz<br>Sample_VEH_27_H3K4me1.fastq.gz<br>Sample_VEH_27_K27me3.fastq.gz<br>Sample_VEH_27_K4me3.fastq.gz<br>Sample_VEH_29_H3K27ac.fastq.gz<br>Sample_VEH_29_H3K4me1.fastq.gz<br>Sample_VEH_29_K27me3.fastq.gz<br>Sample_VEH_29_K4me3.fastq.gz<br>Sample_VEH_33_H3K27ac.fastq.gz<br>Sample_VEH_33_H3K4me1.fastq.gz<br>Sample_VEH_33_K27me3.fastq.gz<br>Sample_VEH_33_K4me3.fastq.gz<br>trim.Sample_10_VEH_2_K4me3.signals.tdf<br>trim.Sample_11_VEH_3_K4me3.signals.tdf<br>trim.Sample_12_VEH_4_K4me3.signals.tdf<br>trim.Sample_16_BPA_2_K4me3.signals.tdf<br>trim.Sample_17_BPA_3_K4me3.signals.tdf<br>trim.Sample_18_BPA_4_K4me3.signals.tdf<br>trim.Sample_19_VEH_2_K4me1.signals.tdf

trim.Sample_20_VEH_3_K4me1.signals.tdf
trim.Sample_21_VEH_4_K4me1.signals.tdf
trim.Sample_25_BPA_2_K4me1.signals.tdf
trim.Sample_26_BPA_3_K4me1.signals.tdf
trim.Sample_27_BPA_4_K4me1.signals.tdf
trim.Sample_28_VEH_2_K27ac.signals.tdf
trim.Sample_29_VEH_3_K27ac.signals.tdf
trim.Sample_BPA_14_H3K27ac.signals.tdf
trim.Sample_BPA_14_H3K4me1.signals.tdf
trim.Sample_BPA_14_K27me3.signals.tdf
trim.Sample_BPA_14_K4me3.signals.tdf
trim.Sample_BPA_23_H3K27ac.signals.tdf
trim.Sample_BPA_23_H3K4me1.signals.tdf
trim.Sample_BPA_23_K27me3.signals.tdf
trim.Sample_BPA_23_K4me3.signals.tdf
trim.Sample_BPA_25_H3K27ac.signals.tdf
trim.Sample_BPA_25_H3K4me1.signals.tdf
trim.Sample_BPA_25_K27me3.signals.tdf
trim.Sample_BPA_25_K4me3.signals.tdf
trim.Sample_VEH_27_H3K27ac.signals.tdf
trim.Sample_VEH_27_H3K4me1.signals.tdf
trim.Sample_VEH_27_K27me3.signals.tdf
trim.Sample_VEH_27_K4me3.signals.tdf
trim.Sample_VEH_29_H3K27ac.signals.tdf
trim.Sample_VEH_29_H3K4me1.signals.tdf
trim.Sample_VEH_29_K27me3.signals.tdf
trim.Sample_VEH_29_K4me3.signals.tdf
trim.Sample_VEH_33_H3K27ac.signals.tdf
trim.Sample_VEH_33_H3K4me1.signals.tdf
trim.Sample_VEH_33_K27me3.signals.tdf
trim.Sample_VEH_33_K4me3.signals.tdf
trim.Sample_10_VEH_2_K4me3.signals.wig.gz
trim.Sample_11_VEH_3_K4me3.signals.wig.gz
trim.Sample_12_VEH_4_K4me3.signals.wig.gz
trim.Sample_16_BPA_2_K4me3.signals.wig.gz
trim.Sample_17_BPA_3_K4me3.signals.wig.gz
trim.Sample_18_BPA_4_K4me3.signals.wig.gz
trim.Sample_19_VEH_2_K4me1.signals.wig.gz
trim.Sample_20_VEH_3_K4me1.signals.wig.gz
trim.Sample_21_VEH_4_K4me1.signals.wig.gz
trim.Sample_25_BPA_2_K4me1.signals.wig.gz
trim.Sample_26_BPA_3_K4me1.signals.wig.gz
trim.Sample_27_BPA_4_K4me1.signals.wig.gz
trim.Sample_28_VEH_2_K27ac.signals.wig.gz
trim.Sample_29_VEH_3_K27ac.signals.wig.gz
trim.Sample_30_VEH_4_K27ac.signals.wig.gz
trim.Sample_30_VEH_4_K27ac.signals.tdf
trim.Sample_34_BPA_2_K27ac.signals.tdf
trim.Sample_35_BPA_3_K27ac.signals.tdf
trim.Sample_36_BPA_4_K27ac.signals.tdf
trim.Sample_37_VEH_2_K27me3.signals.tdf
trim.Sample_38_VEH_3_K27me3.signals.tdf
trim.Sample_39_VEH_4_K27me3.signals.tdf
trim.Sample_43_BPA_2_K27me3.signals.tdf
trim.Sample_44_BPA_3_K27me3.signals.tdf
trim.Sample_45_BPA_4_K27me3.signals.tdf
trim.Sample_BPA_14_H3K27ac.signals.wig.gz
trim.Sample_BPA_14_H3K4me1.signals.wig.gz
trim.Sample_BPA_14_K27me3.signals.wig.gz
trim.Sample_BPA_14_K4me3.signals.wig.gz
trim.Sample_BPA_23_H3K27ac.signals.wig.gz
trim.Sample_BPA_23_H3K4me1.signals.wig.gz
trim.Sample_BPA_23_K27me3.signals.wig.gz
trim.Sample_BPA_23_K4me3.signals.wig.gz
trim.Sample_BPA_25_H3K27ac.signals.wig.gz
trim.Sample_BPA_25_H3K4me1.signals.wig.gz
trim.Sample_BPA_25_K27me3.signals.wig.gz
trim.Sample_BPA_25_K4me3.signals.wig.gz

trim.Sample_VEH_27_H3K27ac.signals.wig.gz
trim.Sample_VEH_27_H3K4me1.signals.wig.gz
trim.Sample_VEH_27_K27me3.signals.wig.gz
trim.Sample_VEH_27_K4me3.signals.wig.gz
trim.Sample_VEH_29_H3K27ac.signals.wig.gz
trim.Sample_VEH_29_H3K4me1.signals.wig.gz
trim.Sample_VEH_29_K27me3.signals.wig.gz
trim.Sample_VEH_29_K4me3.signals.wig.gz
trim.Sample_VEH_33_H3K27ac.signals.wig.gz
trim.Sample_VEH_33_H3K4me1.signals.wig.gz
trim.Sample_VEH_33_K27me3.signals.wig.gz
trim.Sample_VEH_33_K4me3.signals.wig.gz

Genome browser session (e.g. UCSC)

No longer applicable

Methodology

Replicates

Three biological replicates from distinct animals were used per treatment group.

Sequencing depth

Single-end, 37 bp reads were collected, 14-44 million reads per sample were obtained, and 6-25 million uniquely mapped reads were present.

Antibodies

H3K4me3 rabbit polyclonal antibody from Active Motif catalog number 39915; H3K27ac rabbit polyclonal antibody (ChIP grade) from Abcam catalog number ab4729; H3K4me1 rabbit polyclonal antibody (ChIP grade) from Abcam catalog number ab8895; H3K27me3 rabbit polyclonal antibody from Active Motif catalog number 39155

Peak calling parameters

We did not call peaks, we called differential regions using diffReps software v1.55.6.

Data quality

ChIP-seq in rat liver samples yielded 14-44 million single end reads per sample. Reads were trimmed for low quality basepairs using TrimGalore. Data was mapped to the rat genome build UCSC rn6 using the bowtie2 software. Duplicate reads were removed.

Software

Differential ChIP-Seq analysis was performed using diffReps 1.55.6. Heatmaps, PCA and volcano plots were generated using the Python packages matplotlib 2.2.3 and seaborn 0.9.0. Transcriptomic based pathway analysis was carried out using the Python scientific library scipy 1.1.0. Metabolomics pathway analysis were generated using WikiPathways and the Python scientific library scipy 1.1.0. Signal tracks were generated using bedtools 2.27.1 and displayed using Integrated Genome Viewer 2.4.17. Circos plots were generated using circos 0.69-6. ChIP-Seq venn diagrams were created using the Python library matplotlib-venn 0.11.5. Chromatin epigenomic maps were generated using ChromHMM 1.18.
